# Human herpesvirus 6A promotes glycolysis in infected T cells by activation of mTOR signaling

**DOI:** 10.1371/journal.ppat.1008568

**Published:** 2020-06-09

**Authors:** Zhisheng Wu, Junli Jia, Xianyi Xu, Mengyuan Xu, Guangyong Peng, Jingjing Ma, Xuefeng Jiang, Jialin Yao, Kun Yao, Lingyun Li, Huamin Tang

**Affiliations:** 1 Department of Immunology, Nanjing Medical University, Nanjing, P. R. China; 2 Division of Infectious Diseases, Allergy & Immunology and Department of Internal Medicine, Saint Louis University School of Medicine, Saint Louis, Missouri, United States of America; 3 Department of Medical Genetics, Nanjing Medical University, Nanjing, P. R. China; 4 Key Laboratory of Antibody Technique of Ministry of Health, Nanjing Medical University, Nanjing, P. R. China; Wayne State University, UNITED STATES

## Abstract

Human herpesvirus 6 (HHV-6) is an important immunosuppressive and immunomodulatory virus worldwide. However, whether and how HHV-6 infection influences the metabolic machinery of the host cell to provide the energy and biosynthetic resources for virus propagation remains unknown. In this study, we identified that HHV-6A infection promotes glucose metabolism in infected T cells, resulting in elevated glycolytic activity with an increase of glucose uptake, glucose consumption and lactate secretion. Furthermore, we explored the mechanisms involved in HHV-6A-mediated glycolytic activation in the infected T cells. We found increased expressions of the key glucose transporters and glycolytic enzymes in HHV-6A-infected T cells. In addition, HHV-6A infection dramatically activated AKT-mTORC1 signaling in the infected T cells and pharmacological inhibition of mTORC1 blocked HHV-6A-mediated glycolytic activation. We also found that direct inhibition of glycolysis by 2-Deoxy-D-glucose (2-DG) or inhibition of mTORC1 activity in HHV-6A-infected T cells effectively reduced HHV-6 DNA replication, protein synthesis and virion production. These results not only reveal the mechanism of how HHV-6 infection affects host cell metabolism, but also suggest that targeting the metabolic pathway could be a new avenue for HHV-6 therapy.

## Author summary

Human herpesvirus 6 (HHV-6) is a member of the betaherpesvirinae family, which primarily infects T lymphocytes. In the study presented here, we have demonstrated that HHV-6A infection promotes glucose metabolism in infected T cells. Further exploration into the mechanism demonstrated that HHV-6A infection increases the expressions of the key glucose transporters and glycolytic enzymes, as well as activates the AKT-mTORC1 signaling, which is involved in HHV-6A-induced glycolysis activation in HHV-6-infected T cells. Importantly, suppression of glycolysis or mTORC1 activity effectively reduced HHV-6A propagation. Therefore, recognition of the effects of HHV-6 on host cell metabolism will not only facilitate a better understanding of viral pathogenesis but also could reveal potential therapeutic targets for HHV-6-associated diseases by metabolic manipulation.

## Introduction

Human herpesvirus 6 (HHV-6) is a ubiquitous pathogen of the betaherpesvirinae family, with a nearly 90% seroprevalence rate in healthy adults[1, 2]. HHV-6 establishes lifelong latency after the initial productive infection, but unique among different human herpesviruses, HHV-6 has the ability to integrate into human chromosomes[3, 4]. Recently, HHV-6 has been classified into two distinct species, HHV-6A and HHV-6B, with significant differences in their biological, immunological, and pathogenic characteristics[5, 6]. HHV-6B is the causative agent for exanthema subitum in young children[7]. Reactivation of HHV-6B frequently occurs in immunocompromised patients such as solid organ or hematopoietic stem cell transplant recipients. HHV-6 reactivation in transplant recipients is associated with poor outcomes, including acute graft-versus-host disease (GVHD), pneumonia, delayed engraftment, as well as lethal encephalitis[8, 9]. In addition, several studies suggest HHV-6A might be involved in other pathologies, such as multiple sclerosis[10], Hashimoto’s thyroiditis[11, 12], the Alzheimer’s disease[13] and glioma[14].

Understanding how virus infection manipulates cellular metabolism is an emerging and critical field for effective vaccine development and virus treatment. Increasing evidence suggests that both DNA viruses and RNA viruses can modulate the host cell metabolic profiles after their infection[15, 16]. This mechanism also occurs in the infection of human herpesvirus family. Human cytomegalovirus (HCMV) can alter glycolysis, glutaminolysis, and fatty acid synthesis in infected cells, which is required for maximal virus production[17–20]. Kaposi’s sarcoma-associated herpesvirus (KSHV) infection induces a Warburg effect in human endothelial cells (ECs) and lipogenesis in ECs and PEL cells, and these altered metabolic processes are required for maintaining KSHV latency[21, 22]. Epstein-Barr virus-encoded LMP1 significantly increases glycolysis in nasopharyngeal carcinoma (NPC) cells through up-regulation of hexokinase 2 (HK2), and then facilitates proliferation by blocking apoptosis[23]. In addition, herpes simplex virus 1 (HSV-1) has been reported to impact tricarboxylic acid (TCA) cycle metabolic fluxes during infection[24]. However, whether and how HHV-6 regulates the metabolism of host cells to support its replication and/or persistence remains unknown.

In this study, we observed for the first time that HHV-6A infection dramatically promotes glycolysis in infected T cells with an increase in glucose uptake, glucose consumption and lactate production. The expression levels of glucose transporter 1 (Glut1), glucose transporter 3 (Glut3) and several key glycolytic enzymes are up-regulated in HHV-6A infected cells. Furthermore, we have demonstrated that HHV-6A infection activates AKT-mTORC1 signaling, which is involved in HHV-6A-mediated glycolysis activation in T cells. In addition, inhibition of glycolysis by 2-DG or pharmacologically inhibiting mTORC1 activity dramatically reduced viral DNA replication, protein synthesis and infectious virion production.

## Results

### HHV-6A infection increases glucose metabolism and glycolysis in infected T cells

**Fig 1:**
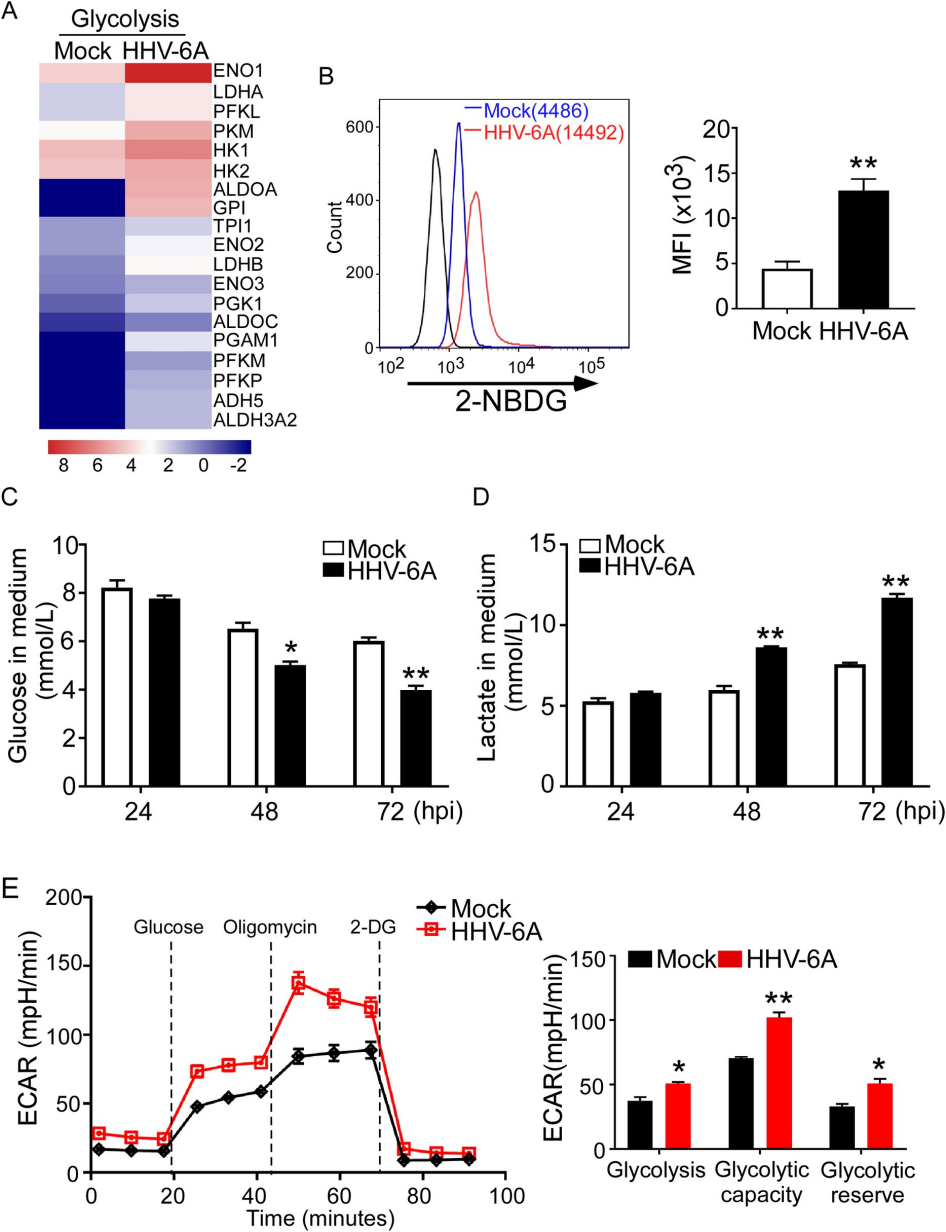
HHV-6A infection increases glucose metabolism and glycolysis in HSB-2 cells. **(A)** Alterations of genes involved in glycolysis were identified and ranked in HSB-2 cells after infection with HHV-6A. Human HSB-2 cells were infected with HHV-6A for 72 hours. Total RNA was purified and pooled, and transcriptome analyses were performed by RNA sequencing. (**B)** HHV-6A infection significantly increased glucose uptake in HSB-2 cells. HHV-6A infected and mock infected HSB-2 cells were cultured for 72 hours, glucose uptake was determined by flow cytometry after addition of 2-NBDG for 15 min. Results in the right histogram are mean ± SD of fluorescence intensity (MFI) quantifications from three independent experiments. **p < 0.01, compared with mock infection groups. **(C)** HHV-6A infection decreased glucose levels in the culture medium of in HSB-2 cells. HHV-6A infected and mock infected HSB-2 cells were cultured for 24 h, 48 h and 72 h, and glucose levels in the culture medium were determined using a Glucose Oxidation Assay Kit. Results shown in histogram are mean ± SD from three independent experiments. *p<0.05 and **p<0.01, compared with the respective mock infection groups. **(D)** HHV-6A infected HSB-2 cells produce more lactate than mock-infected cells. The lactate levels in the culture supernatants were determined by the lactate assay kit. Results shown are mean ± SD from three independent experiments. **p<0.01, compared with the levels in mock infected cells. (E) ECAR in mock-infected and HHV-6A-infected cells was measured using extracellular flux analysis. Mock- and HHV-6-infected HSB-2 cells were cultured for 24 h. The ECAR over time was measured at a basal level and after the injection of glycose, oligomycin (an inhibitor of ATP synthase) and 2-DG (an inhibitor of glycolysis). Statistical analysis of glycolysis, glycolytic capacity and glycolytic reserve are shown on the right. Data are mean ± SD of triplicates * p < 0.05, **p < 0.01, compared with the levels in mock infected cells.

HSB-2, a T-lymphoblastoid cell line, permissive to HHV-6 infection, was used in this study. Initially, efficient infection of HSB-2 cells with HHV-6A was confirmed by observation of cytopathic effects (CPE), immunofluorescence and western blot assay (**S1A–S1C Fig**). To determine if HHV-6A infection affects cell metabolism of host cells, we conducted a global RNA sequencing analysis of changes in whole genome expression in HHV-6 infected cells compared with mock-infected cells. We found that metabolism-related genes were significantly up-regulated in virus-infected cells. In particular, key glycolytic enzyme genes were markedly increased in HHV-6A infected cells, suggesting heightened glycolytic activity (**Fig 1A**). To further confirm this result, we first examined the glucose uptake ability of HSB-2 cells infected with HHV-6A, using a fluorescent glucose analog 2-NBDG (2-(N-(7-Nitrobenz-2-oxa-1, 3-diazol-4-yl) Amino)-2-Deoxyglucose) labeling assay. We found that the uptake of 2-NBDG was increased in HHV-6A-infected HSB-2 cells compared with mock-infected cells at 72 h post-infection, suggesting that the glucose uptake was increased (**Fig 1B**). We then determined the glucose consumption in HHV-6A infected cells. As shown in **Fig 1C**, the glucose concentration decreased much faster over time in culture medium of HHV-6A-infected HSB-2 cells than that in mock-infected cells. To determine if the increased glucose uptake of HHV-6-infected HSB-2 cells leads to increased glycolysis, we further examined the production of glycolysis metabolite lactic acid. As shown in **Fig 1D**, there is a ~50% increase (mock 7.57±0.11mmol/L versus HHV6 11.69±0.34 mmol/L) in lactic acid secretion in HHV-6A-infected cells compared with that in mock-infected cells at 72 h post-infection. Additionally, the extracellular acidification rate (ECAR), a marker of glycolysis were directly measured using the Seahorse XF extracellular flux analyzer. As shown in **Fig 1E**, HHV-6A-infected HSB-2 cells showed significantly increased ECARs following the addition of glucose compared with those of mock-infected cells. Then, oligomycin was added to inhibit mitochondrial ATP production and induce maximal glycolysis. Importantly, HHV-6-infected cells showed robust increases in the ECAR levels following oligomycin treatment, compared with mock-infected cells. Finally, ECARs were dramatically reduced both in HHV-6-infected cells and mock-infected cells following the administration of the glycolysis inhibitor 2-deoxy-D-glucose (2-DG). In conclusion, glycolysis, glycolytic capacity and glycolytic reserve were all substantially increased in HHV-6-infected cells in comparison with these parameters in mock-infected cells. Collectively, these results clearly demonstrate that HHV-6A infection increases glucose metabolism in HSB-2 cells.

### The expression levels of Glut1 and Glut3 are significantly increased in HHV-6A infected T cells

**Fig 2:**
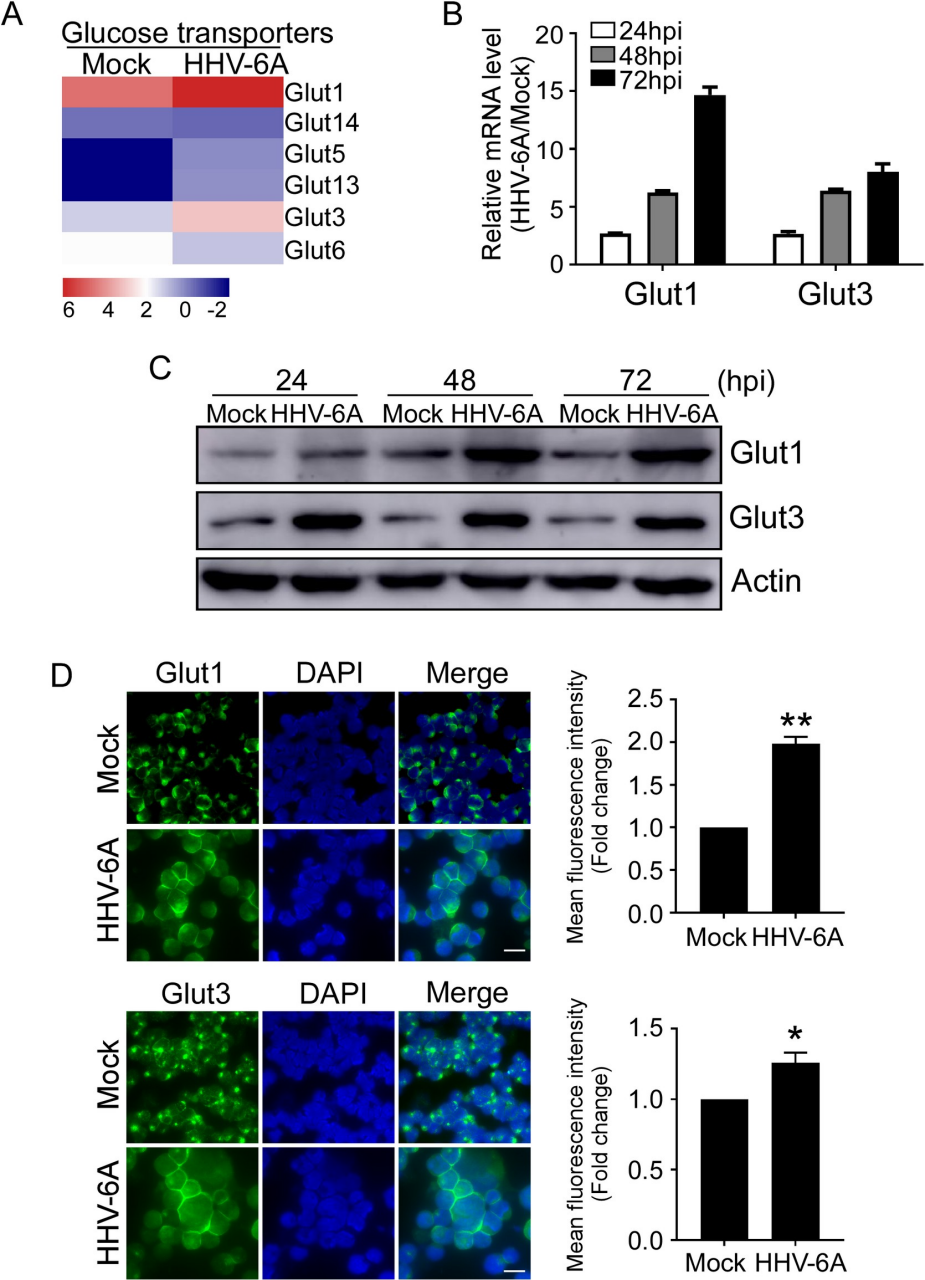
HHV-6A infection induces increased Glut1 and Glut3 expression. **(A)** RNA sequencing analysis of glucose transporter genes expression in in HSB-2 cells after infection with HHV-6A. Human HSB-2 cells were infected with HHV-6A for 72 hours. Total RNA was purified and pooled, and transcriptome analyses were performed by RNA sequencing analysis. **(B)** HHV-6A infection significantly increased the mRNA levels of Glut1 and Glut3 in HSB-2 cells. HSB-2 cells were mock infected or infected with HHV-6A. The total RNA was isolated at 24, 48, and 72 hpi and then mRNA levels were analyzed by quantitative RT-PCR. The expression levels of each gene were normalized to β-actin and plotted with respect to mock infection. Data shown are mean ± SD from three independent experiments. **(C)** HHV-6A infection up-regulated protein expressions of Glut1 and Glut3 in HSB-2 cells. Whole-cell extracts were prepared from mock-infected and HHV-6A-infected infected HSB-2 cells at 24, 48 and 72 hpi. Glut1, Glut3 and β-actin protein levels were determined by Western analysis. **(D)** HHV-6A infection induced Glut1 and Glut3 expression and translocation. Glut1 and Glut3 expression were determined by an indirect immunofluorescence assay. Mock and HHV-6A-infected HSB-2 cells were stained for nuclear DNA (DAPI, blue) and Glut1 and Glut3 (green) antibodies at 48 h post-infection. Scale bar, 10 μm. The results in the right of the histograms are mean ± SD of fluorescence intensity (MFI) quantifications of Gluts from three independent experiments. * p<0.05, **p<0.01, compared with the mock-infected group.

The glucose transporter (Glut) family is primarily responsible for the uptake of glucose into cells[25, 26]. It was reported that Glut1-mediated glucose transport is required for efficient HIV-1 infection of CD4^+^ T cells and thymocytes[27]. We next determined whether the increase in glucose uptake and glucose consumption in HHV-6A-infected HSB-2 cells is due to alterations of Gluts. RNA sequencing analyses suggested apparent changes in Glut gene expression in HHV-6A-infected cells (**Fig 2A**). To confirm the RNA sequencing results, we examined the mRNA transcript abundance of the Glut transporter family in HSB-2 cells. It was found that mRNA levels of Glut1 and Glut3 were much higher than other Glut family members in HSB-2 cells (**S2 Fig**). We therefore examined the transcription levels of Glut1 and Glut3 in mock-infected and HHV-6A infected HSB-2 cells using real-time PCR. As expected, HHV-6A infection significantly increased the mRNA levels of Glut1 and Glut3 in HSB-2 cells at 24 h post-infection, and the expression level progressively increased at 48 h and 72 h post infection (more than a 10-fold increase at 72 h post infection compared with mock-infected cells) (**Fig 2B**). In addition, we examined the protein levels of Glut1 and Glut3 by the Western blot analysis. Consistent with the mRNA expression levels, the protein levels of Glut1 and Glut3 were also increased in HHV-6A-infected cells compared with mock-infected cells (**Fig 2C**).

To be functional, the glucose transporters must be located to the cell membrane to uptake glucose into the cells[25, 26]. Several reports have shown that virus infection could induce translocation of the Glut1 molecule to the cell membrane[28, 29]. We thus hypothesized that HHV-6A infection may also alter Gluts translocation. We determined how Gluts localize and traffic in HHV-6A-infected HSB-2 cells using an immunofluorescence analysis. As shown in **Fig 2D**, we observed that HHV-6A-infected cells exhibited high fluorescence intensities in cell boundaries compared with mock-infected cells, suggesting a fairly dramatic relocalization of Glut1 and Glut3. Collectively, these results indicate that HHV-6A infection not only increases Glut expression but also promotes the transporters translocation.

### HHV-6A infection increases key glycolytic enzymes expression

**Fig 3:**
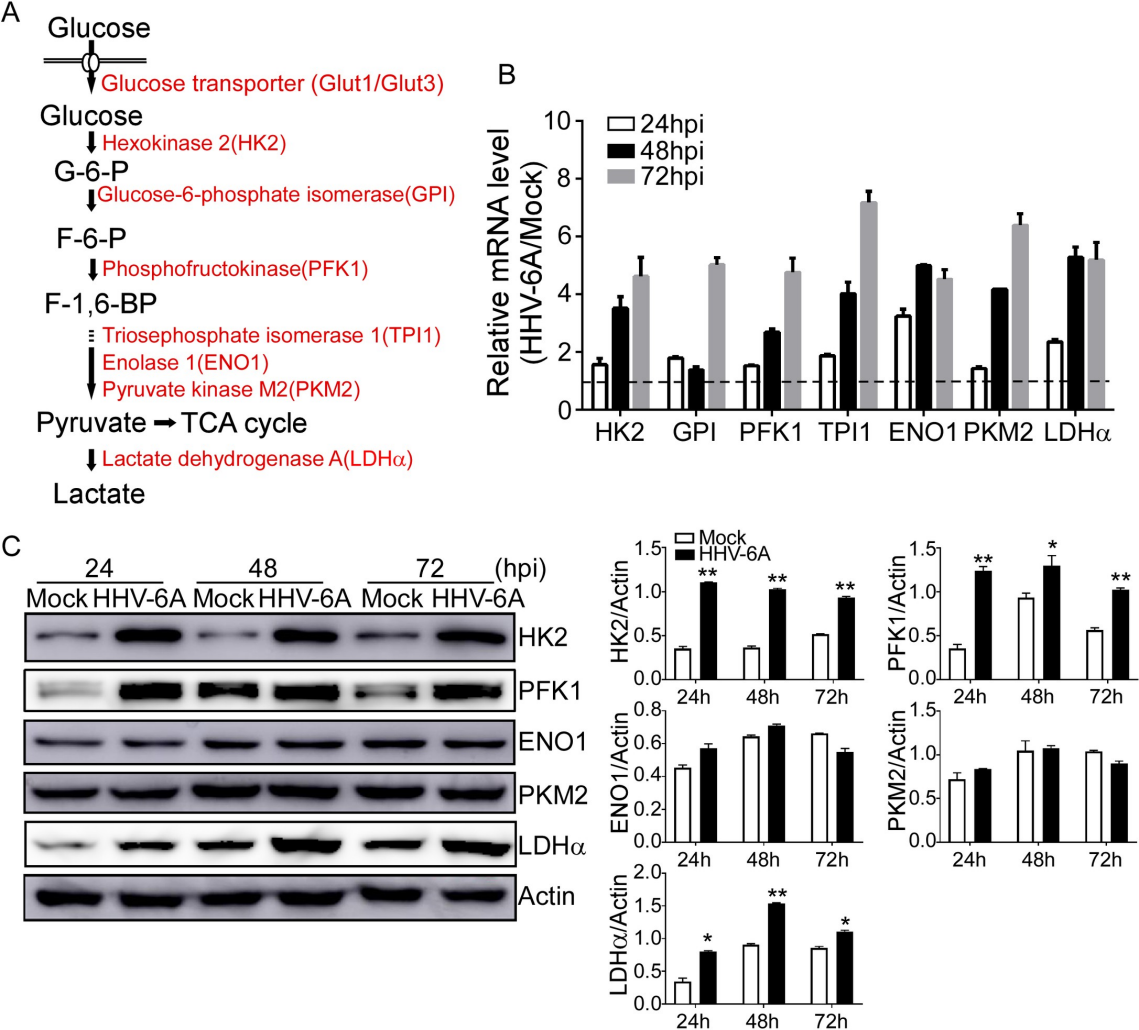
HHV-6A infection increases glycolytic enzyme expression. **(A)** A schematic diagram of the glycolysis pathway. The key glucose transporters and glycolytic enzymes are shown in red. **(B)** HHV-6A infection significantly up-regulated mRNA levels of key glycolytic enzymes in HSB-2 cells. HSB-2 cells were mock infected or infected with HHV-6A. The total RNA was isolated at 24, 48, and 72 hpi and then mRNA levels were analyzed by quantitative PCR. The expression levels of each gene were normalized to β-actin and plotted with respect to mock infection. Data shown are mean ± SD from three independent experiments. **(C)** Protein levels of the key glycolytic enzymes in mock-infected or HHV-6A-infected HSB-2 cells. HSB-2 cells were mock infected or infected with HHV-6A. (Left) Cells were harvested for protein analysis at 24, 48, and 72 hpi and processed for Western analysis using the indicated antibodies. (Right) Quantification of glycolytic enzymes levels during HHV-6A infection. The glycolytic enzymes expression levels were quantitatively analyzed and compared with β-actin expression with a densitometer. Results are means ± SD from three independent experiments. *p<0.05, **p<0.01, compared with the mock infected group.

Considering that the increase in glucose consumption and lactate production were observed in HSB-2 cells during HHV-6A infection, we reasoned that the expression of glycolytic enzymes might be up-regulated by infection. Our transcriptome analyses demonstrated that HHV-6A infection significantly increased the gene expression levels of enzymes involved in glycolysis in HHV-6A infected cells (**Fig 1A**). We then analyzed the mRNA expression levels of a panel of key glycolytic enzymes using quantitative real-time PCR, including glycolysis-related enzymes hexokinase 2 (HK2), glucose-6-phosphate isomerase (GPI), phosphofructokinase 1 (PFK1), triosephosphate isomerase 1 (TPI1), enolase 1 (ENO1), pyruvate kinase muscle 2 (PKM2) and lactate dehydrogenase A (LDHα) (**Fig 3A**). We found that HHV-6A infection markedly increased the expression of the glycolytic enzymes in HHV-6A infected cells compared with mock-infected cells in a time-dependent manner (**Fig 3B**). We next examined the protein levels of those glycolytic enzymes using the Western blot analysis. As shown in **Fig 3C**, HHV-6A infection strongly induced the protein levels of HK2, PFK1, and LDHα in HSB-2 cells compared with the mock-infected cells at 48 and 72 h post-infection. However, HHV-6A had no obvious effect on ENO1 and PKM2 expression. These results suggest that HHV-6A-induced up-regulation of several glycolytic enzymes might be part of the molecular basis responsible for the HHV-6A-mediated enhanced glycolysis. In addition, we determined the mRNA levels of key enzymes involved in canonical TCA cycles by real-time PCR analysis and found that several TCA cycle enzymes expression were also increased in the HHV-6A-infected cells compared with mock-infected cells **(S3 Fig)**.

### HHV-6A infection activates AKT-mTORC1 signaling in HSB-2 cells

**Fig 4:**
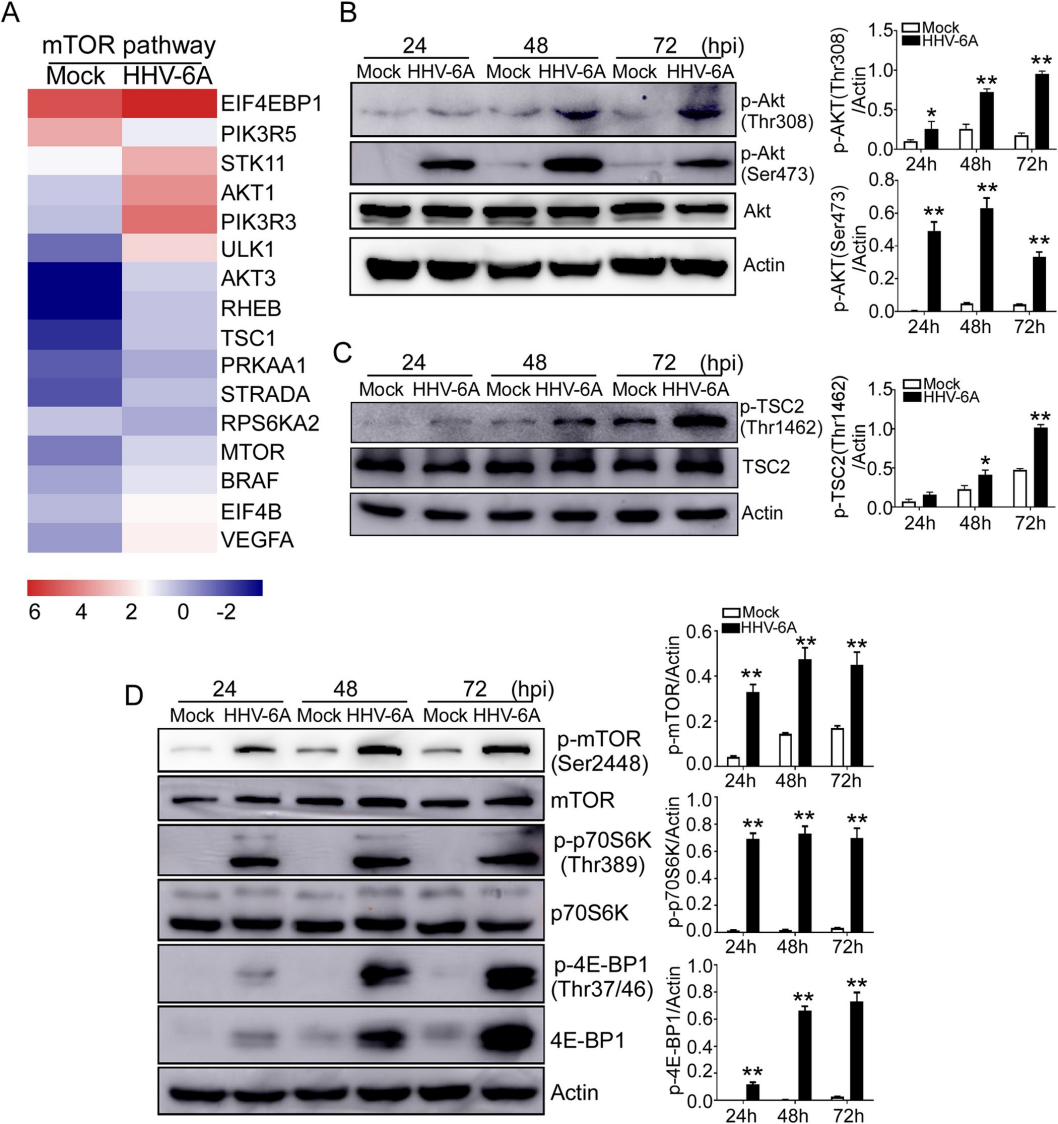
HHV-6A infection induces activation of the AKT- mTORC1 signaling pathway. **(A)** Alterations of genes involved in mTOR signaling pathway were identified and ranked in HSB-2 cells after infection with HHV-6A. Human HSB-2 cells were infected with HHV-6A for 72 hours. Total RNA was purified and pooled, and transcriptome analyses were performed by RNA sequencing analysis. **(B)** HHV-6A infection induced phosphorylation of AKT in infected HSB-2 cells. Mock infected and HHV-6A infected cells were lysed and analyzed by western blotting using specific antibodies against Akt, p-Akt (Thr308) and p-Akt (Ser473). Phosphorylated AKT protein levels were quantitatively analyzed and were compared with β-actin expression with a densitometer. Results are means ± SD from three independent experiments. **p<0.01, compared with the mock-infected group. **(C)** HHV-6A infection induced phosphorylation of TSC2 in infected HSB-2 cells. Mock infected and HHV-6A infected cells were lysed and analyzed by western blotting using specific antibodies against TSC2 and p-TSC2 (Thr1462). Phosphorylated TSC2 protein levels were quantitatively analyzed and were compared with β-actin expression with a densitometer. Results are means ± SD from three independent experiments. *p<0.05, **p<0.01, compared with the mock-infected group. **(D**) HHV-6A infection induces activation of the mTORC1 signaling pathway in infected HSB-2 cells. Mock and HHV-6A-infected cells were lysed and analyzed by Western blotting using antibodies against various proteins involved in mTORC1 signaling. P-mTOR, p-p70S6K and p-4E-BP1 protein levels were quantitatively analyzed and were compared with β-actin expression with a densitometer. Results are means ± SD from three independent experiments. **p<0.01, compared with the mock-infected group.

Mammalian target of rapamycin (mTOR) signaling plays a critical role in the regulation of energy metabolism, cell growth and proliferation[30, 31]. RNA sequencing analyses showed that HHV-6A infection induced significant alterations in 16 genes involved in mTOR signaling in infected HSB-2 cells (**Fig 4A**). Knowing that PI3K/AKT is a well-documented upstream site of mTORC1 signaling, which is involved in activation of mTORC1[32, 33], we next determined AKT expression and phosphorylation levels in HHV-6A infected cells. As shown in **Fig 4B**, HHV-6A infection effectively induced phosphorylation of AKT at Thr308 and Ser473 in virus-infected cells. In contrast, the phosphorylation of AKT expression was hardly detected in the mock-infected cells at various times. Consistent with our results, it was reported that HCMV infection is able to activate AKT signaling, which was shown by the increase in AKT phosphorylation at Ser473[34]. Given that tuberous sclerosis complex (TSC), a negative regulator of mTORC1, is located downstream of AKT[35, 36], we investigated TSC2 (a major component of TSC) expression and its inhibitory phosphorylation levels in HHV-6A infected cells. And we found that HHV-6A infection significantly induced TSC2 phosphorylation at Thr1462 (the major AKT-dependent phosphorylation site), indicating inactivation of TSC during HHV-6 infection (**Fig 4C).**

We next determined whether mTOR signaling is also activated following HHV-6A infection. We analyzed the expression and phosphorylation status of mTOR and its downstream substrates p70S6K and 4E-BP1 in HHV-6A infected T cells. As expected, we found that HHV-6A infection significantly induced phosphorylation of mTOR (Ser2448) and mTORC1 downstream targets p70S6K (Thr389) in HHV-6A infected cells compared with the mock-infected cells. Furthermore, we found that both total 4E-BP1 and phosphorylated 4E-BP1 (Thr37/46) were induced in cells by HHV-6A infection, while there was only minimal expression in the mock-infected cells (**Fig 4D**). In support of the above results, we also found that the phosphorylation of AMPK, a kinase that inhibits mTORC1 signaling[37, 38], was significantly down-regulated following HHV-6A infection (**S4 Fig**). These data clearly indicate that mTORC1 signaling is significantly activated following HHV-6 infection.

### Inhibition of mTORC1 signaling blocks HHV-6A-induced glycolytic activation

**Fig 5:**
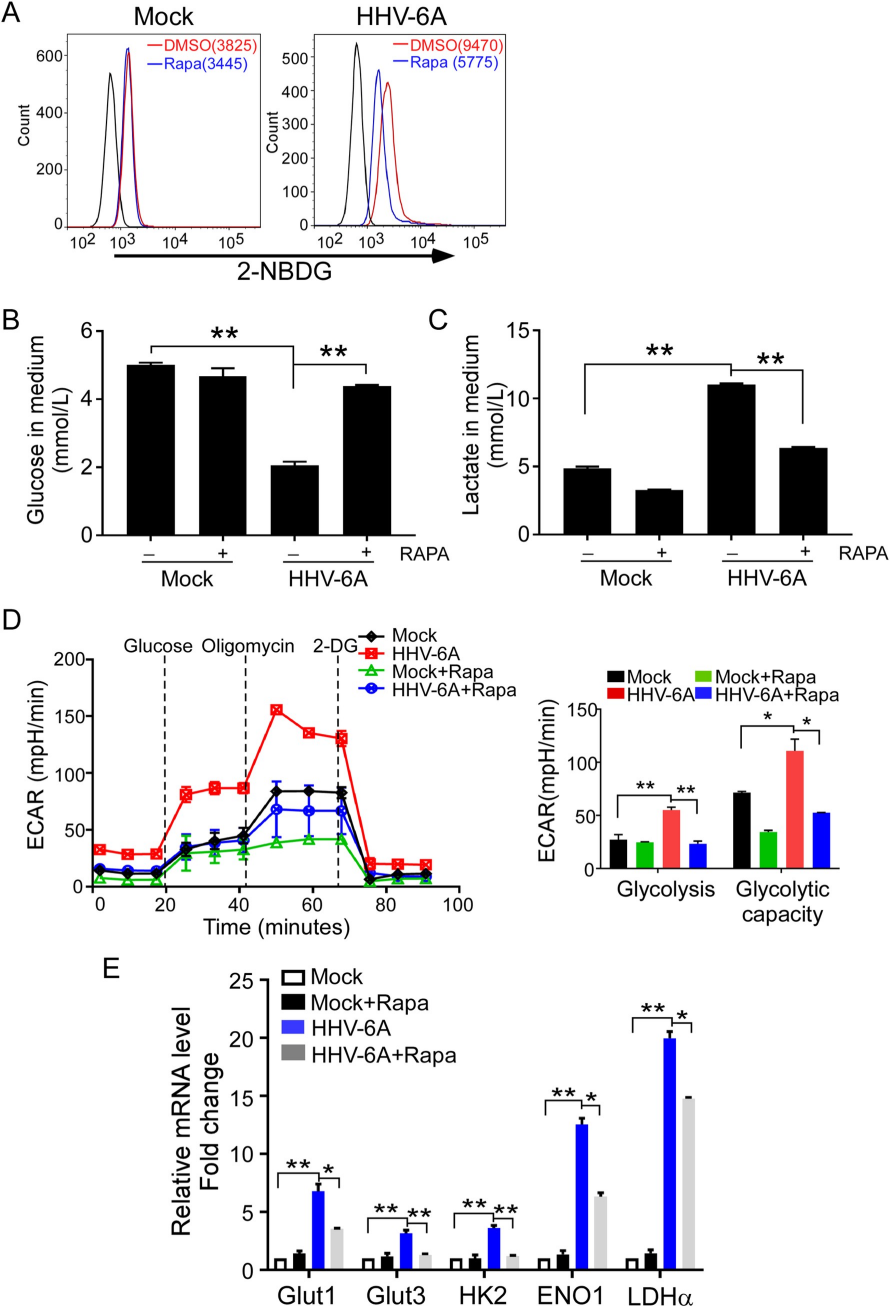
Inhibition of mTOR blocks HHV-6A-mediated glycolytic activation. HSB-2 cells were mock infected or infected with HHV-6A. After adsorption, cells were treated with the mTOR inhibitor rapamycin (200 nM) or DMSO. **(A)** Rapamycin treatment significantly decreased glucose uptake in HHV-6A-infected cells. Glucose uptake was determined by flow cytometry with addition of 2-NBDG for 15 min after 72 h culture. **(B)** Rapamycin treatment increased glucose levels in the culture medium in HHV-6A infected HSB-2 cells. The glucose levels in the culture medium were determined at 72 h post infection using a Glucose Oxidation Assay Kit. Results shown in histogram are mean ± SD from three independent experiments. ** p<0.01, compared with the indicated control group. **(C)** Rapamycin treatment decreased lactate secretion of HSB-2 cell. The lactate levels in culture supernatant was analyzed at 72 h post infection. Results shown in histogram are mean ± SD from three independent experiments. ** p<0.01, compared with the indicated control group. **(D)** Rapamycin treatment decreased ECAR levels in HHV-6A infected HSB-2 cells. Mock- and HHV-6-infected HSB-2 cells were cultured with or without rapamycin for 24 h. The ECAR over time was measured at a basal level and after the injection of glycose, oligomycin (an inhibitor of ATP synthase) and 2-DG (an inhibitor of glycolysis). Statistical analysis of glycolysis and glycolytic capacity are shown on the right. Data are mean ± SD of triplicates *P < 0.05, **P < 0.01, compared with the indicated control group. **(E)** Real-time PCR quantification of Gluts and glycolytic enzymes expression in mock and HHV-6A infected HSB-2 cells with rapamycin treatment for 72 h. Total RNA was isolated from the HSB-2 cells and analyzed by real-time PCR. The expression levels of each gene were normalized to β-actin expression levels and adjusted to the levels in mock-infected HSB-2 cells (served as 1). Results in histogram are the mean ± SD from three independent experiments. * p<0.05, ** p<0.01, compared with the indicated control group.

To further determine whether mTORC1 signaling is important for HHV-6A-mediated activation of glycolysis, we treated HHV-6-infected cells with rapamycin, a specific inhibitor of mTORC1, and then determined glucose metabolism and lactate production. Consistent with the results shown in **Fig 1B**, HHV-6A-infected cells had a significantly higher glucose uptake than that of mock-infected cells (**Fig 5A**). The glucose uptake was markedly decreased in HHV-6-infected cells after rapamycin treatment. Furthermore, rapamycin treatment only had a slight impact on glucose uptake in mock-infected HSB-2 cells (**Fig 5A**). Next, the involvement of mTORC1 signaling in glucose consumption and lactate production in HHV-6A infected HSB-2 cells was explored. We found that rapamycin treatment significantly inhibited glucose consumption in HHV-6A-infected cells. However, in mock-infected HSB-2, there was no obvious difference in glucose consumption with or without rapamycin treatment (**Fig 5B**). In addition, treatment with rapamycin dramatically reduced lactate production in both mock-infected cells and HHV-6-infected cells. However, the inhibition effect was much stronger in HHV-6-infected HSB-2 cells than mock-infected HSB-2. Specifically, in mock-infected cells, rapamycin treatment only slightly decreased lactate production from 4.87±0.199 mmol/L to 3.27±0.031 mmol/L. However, in HHV-6A-infected cells, rapamycin treatment significantly decreased lactate production from 11.02±0.145 mmol/L to 6.38±0.091mmol/L. (**Fig 5C**). In addition, the ECAR levels was also measured following rapamycin treatment both in mock-infected and HHV-6-infected cells. Consistent with the results shown in **Fig 1E**, HHV-6A-infected cells had a significantly higher glycolysis and glycolytic capacity than that of mock-infected cells (**Fig 5D**), and these two parameters were both markedly decreased in HHV-6-infected cells after rapamycin treatment. We also found that the glycolytic capacity decreased in mock-infected cells with rapamycin treatment, which might be due to the toxic effect of rapamycin on these cells.

To investigate further the mechanism of HHV-6A-mediated glycolytic activation, we assessed the impact of mTOR inhibition on virally-induced up-regulations of glucose transporters and glycolytic enzymes. Consistent with the results above, we found that the mRNA levels of Glut1, Glut3, HK2, ENO1, LDHα were dramatically increased in HHV-6A infected cells. However, rapamycin treatment significantly reduced these molecules expression in HHV-6A infected cells (**Fig 5E**). These results clearly suggest that the mTORC1 activation is critical to regulating HHV-6A-mediated glycolytic activation in the infected T cells.

### Pharmaceutical inhibition of glycolysis or mTORC1 activity attenuates HHV-6A viral replication

**Fig 6:**
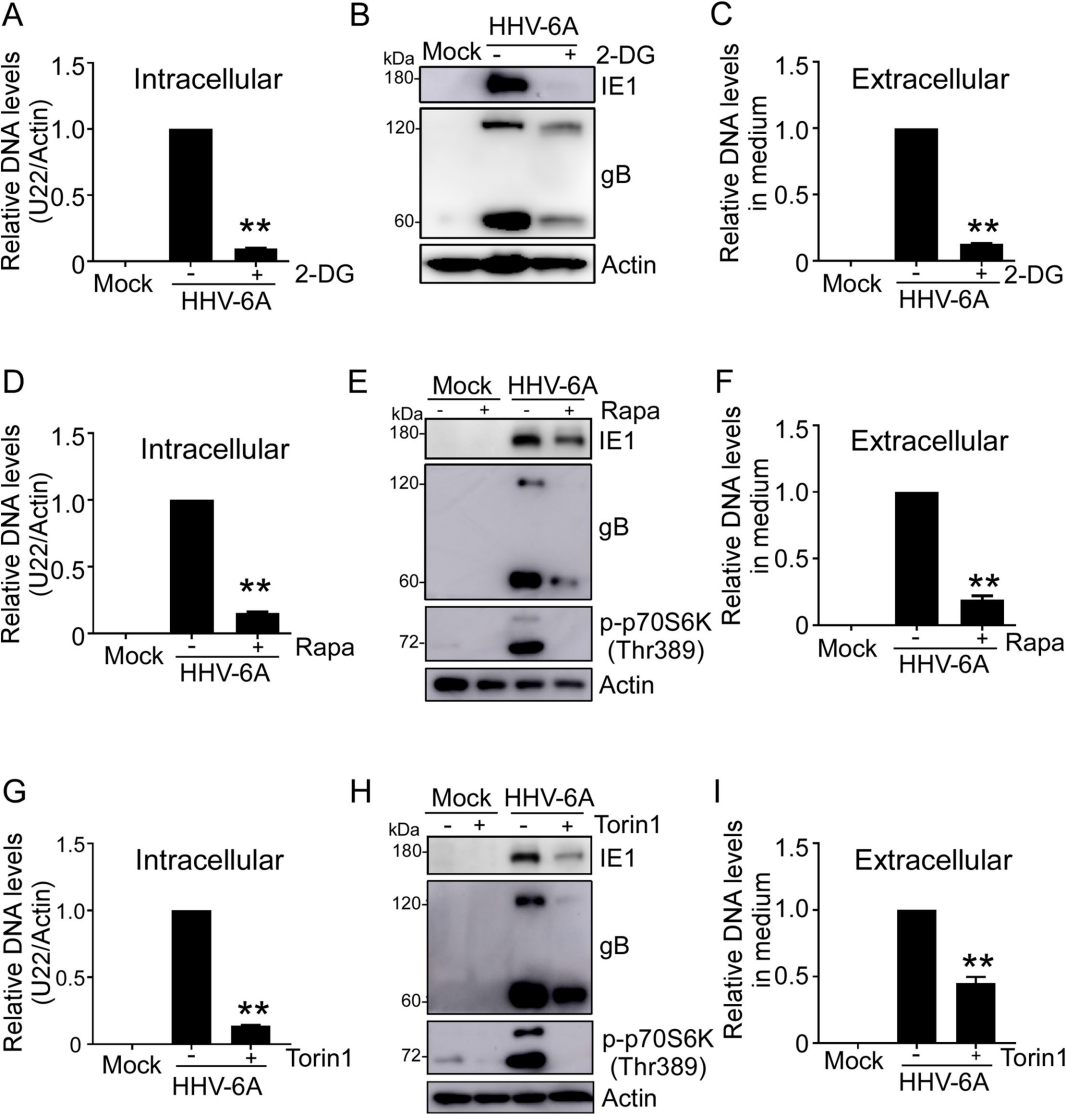
Impact of glycolytic inhibition and mTORC1 inhibition on viral protein and DNA accumulation. **(A), (D) and (G)** Real-time PCR quantification of viral DNA accumulation in HHV-6A infected cells treated with 2-DG, rapamycin or Torin. The HSB-2 cells were mock infected or infected with HHV-6A. After adsorption, cells were treated with 2-DG (1 mM) in A, the mTOR-specific inhibitor rapamycin (100 nM) in D, or Torin1 (5 nM) in G, respectively. Viral DNA was extracted from cells that were harvested at 72 h post infection and the amount of viral U22 gene was determined by real-time PCR analysis. Expression levels of U22 gene were normalized to β-actin expression levels and adjusted to the levels in HHV-6A infected cells (served as 1). Values are means ± SD from three independent experiments. ** p<0.01, compared with the control no inhibitor treatment group. **(B)**, **(E) and (H)** Western blot analysis of viral protein accumulation in HHV-6A-infected cells treated with 2-DG, rapamycin or Torin1. Viral immediate-early protein IE1 and late protein gB were detected by Western blotting using corresponding antibodies at 24 h or 72 h post infection, respectively. **(C), (F) and (I)** Real-time PCR quantification of viral progeny production in the supernatant of HHV-6A infected cell treated with 2-DG, rapamycin or Torin1. At 72 h post infection, the viral DNA from supernatant was extracted and the amount of viral U22 gene was determined by real-time PCR. Expression levels of U22 gene in HHV-6A infected cells served as 1.Values are means ± SD from three independent experiments. ** p<0.01, compared with the untreated group.

Based on our findings that HHV-6A infection increases glycolytic rate in infected HSB-2 cells, we wanted to understand if direct blockage of glycolysis could attenuate viral replication. We performed quantitative PCR and Western blot analyses to determine viral DNA replication and protein expression. The inhibition of glycolysis in HSB-2 cells was achieved with 2-Deoxy-D-glucose (2-DG), a glucose analog that inhibits the conversion of glucose to glucose-6-phosphate by competing with glucose for binding to HK2. We observed that 2-DG treatment significantly reduced glucose uptake, glucose consumption and lactate production in HHV-6A infected cells (**Fig 5A–5C**). Furthermore, treatment of HHV-6A infected cells with 2-DG markedly attenuated viral DNA replication and immediate-early and late protein expression **(Fig 6A and 6B)**. In addition, we tested if 2-DG treatment could reduce virion progeny release by analysis of viral DNA contents in cell supernatant using real-time PCR. As shown in **Fig 6C**, 2-DG treatment significantly reduced the viral DNA content in the cell culture medium compared with the DMSO treated group.

Given that mTORC1 signaling activation is important for HHV-6A-mediated enhanced glycolysis, we determined if mTORC1 signaling inhibition could attenuate virus production similar to that observed with 2-DG treatment. As shown in **Fig 6D and 6E**, there was a significant decrease in viral DNA levels and protein expression in HHV-6A infected cells after rapamycin treatment. We tested if rapamycin treatment could reduce virion progeny release by analysis of viral DNA contents in cell supernatants using real-time PCR. As shown in **Fig 6F**, rapamycin treatment significantly reduced the viral DNA content in cell culture medium compared with the DMSO treated group. We also observed that inhibition of mTOR signaling in HSB-2 cells with Torin1, another mTOR inhibitor that blocks both mTORC1 and mTORC2 complexes, dramatically blocked virus DNA replication, protein expression and virus virion production, which were similar to rapamycin treatment (**Fig 6G–6I)**.These results collectively suggest that both glycolysis and mTORC1 activity is important for HHV-6A efficient replication in host cells.

## Discussion

HHV-6 was initially isolated from patients with lymphoproliferative disorders[39]. Like other herpesviruses, HHV-6 is capable of persisting in the host after primary infection and hardly cleared from its host. Reactivation of HHV-6 frequently occurs in the immunocompromised patients suffering from HIV infection, cancer or following transplantation, which may cause severe consequences[2]. Both HHV-6A and HHV-6B can infect several types of immune cells, either productively or nonproductively. The primary target for productive infection is the activated CD4^+^ T lymphocytes[40], which causes significant phenotypic and functional alterations in the infected cells following HHV-6 infection. In infected CD4^+^ T cells, HHV-6 induces apoptosis[41, 42], inhibits IL-2 synthesis[43] and suppresses T-cell proliferation by cell cycle arrest[44], as well as down-regulates the expression of TCR and MHC-I molecules[45, 46]. So far, no study relating to the effects on cellular metabolism regulation by HHV-6 infection has been reported. In the current study, we investigated for the first time the molecular process by which HHV-6A induces cell metabolism perturbation in the infected T cells, which may be one of the key mechanisms responsible for HHV-6 successful infection and latency establishment in host cells.

Viruses are obligate parasites that depend on host cells to provide energy and molecular precursors for their successful infection. Manipulation of host cell metabolism is a common strategy utilized by many viruses for their infection and persistence[47, 48]. The first landmark study of the viral effect on host metabolism was performed in HCMV infected cells. This study found changes in many metabolic pathways in HCMV infected cells including glycolysis and fatty acid synthesis[17]. Subsequently, many other viruses have been widely studied, including HSV-1, HCV, KSHV, EBV, and Dengue virus[15, 16, 49]. In this study, we demonstrate that HHV-6 infection significantly increases glucose uptake and lactic acid production in infected T cells, indicating glycolysis activation. Furthermore, we found the expression levels of glucose transporters (Glut1 and Glut3) and glycolytic enzymes (such as HK2, PFK1 and LDHα) were significantly increased in HHV-6 infected cells. In support of our findings, several studies with other viruses have shown that virus infection could activate glycolysis using different manners. It has been reported that HSV-1 infection activates glycolysis through up-regulating the expression and activity of phosphofructokinase-1 (PFK-1) in infected cells[50]. EBV LMP1 prompts aerobic glycolysis by activation of the FGFR1 signaling pathway[51]. More recently, it was shown that KSHV employs specific viral microRNAs targeting the key regulators of glucose metabolism and mitochondrial biogenesis in the infected cells to induce glycolytic activity[52]. Adenovirus E4ORF1 can induce MYC activation and subsequently result in elevated expression of specific glycolytic enzymes in infected cells, which is sufficient to promote glucose metabolism of host cells for virus replication[53]. These studies suggest that glycolysis may be a common metabolic pathway explored by many viruses to support virus propagation[20, 49, 53, 54].

Glucose transporters mediate the transport of monosaccharides, polyols and other small carbon compounds across the membranes of eukaryotic cells[55]. In this study, we found that Glut1 and Glut3 expression is much higher than other glucose transporters, and further increased after HHV-6 infection. However, the mRNA transcripts of Glut2 and Glut4 are hardly detected in transcriptional levels in HSB-2 cells. Unlike HHV-6 infection, HCMV infection was shown to dramatically increase the expression of the adipose tissue-specific Glut4 and eliminate the expression of the ubiquitously distributed Glut1 in infected fibroblasts[20]. Glut3 is generally considered as a neuronal glucose transporter, which has been detected in the peripheral cells[56, 57]. Glut3 has both a higher affinity for glucose and greater transport capacity than other the facilitative glucose transporters[58]. In this study, there is an obviously elevated expression of Glut1 and Glut3 in HHV-6A-infected HSB-2 cells, thus enhancing the cell capacity to transport glucose and meet increased energy demands following virus infection.

The glycolytic pathway consists of a series of catalytic reactions which controlled by a number of enzymes, e.g. hexokinase, phosphofructokinase and pyruvate kinase. In this study, we found that HHV-6 enhances glycolysis through upregulation of key glycolytic enzymes HK2 and PFK1 which are the first and third enzymes along the pathway and also the key rate-limiting enzymes that limit the overall glycolytic rate[59, 60]. We also found that HHV-6A have no obvious effect on the expression of ENO1 and PKM2 which control the final steps of glycolysis. PKM2 is the rate-limiting enzyme in the last stage of the glycolytic pathway and when its catalytic rate is lower than the upstream glycolytic rate, phosphoenolpyruvate (PEP) and its upstream glycolytic intermediates may accumulate for the synthesis of macromolecules[61]. Thus, we speculate that during HHV-6 infection there will be an accumulation of glycolytic intermediates which can be used for the biosynthesis of lipids, nucleotides, and proteins required for rapidly virus reproduction.

mTOR is a highly conserved serine/threonine kinase that controls cell growth, cell proliferation and energy metabolism[62, 63]. In this study, we demonstrated that HHV-6A infection dramatically promotes the phosphorylation levels of AKT, TSC2, mTORC1, P70S6K and 4E-BP1, all indicating mTORC1 activation. Additional studies demonstrated that inhibition of mTORC1 activity using a specific pharmaceutical inhibitor, rapamycin, not only decreases glycolysis in the infected T cells but also inhibits virus replication. In molecular levels, it was shown that the key glycolytic genes (Glut1, Glut3, HK2, ENO1 and LDHα) expression are down-regulated in HHV-6A infected cells after rapamycin treatment. From these data, we concluded that HHV-6A-induced glycolysis activation is mainly depended on AKT-mTORC1 signaling activation, resulting in up-regulation of glucose transporters and glycolytic enzymes expression, and subsequently elevating glycolysis in infected cells. In support of our assertion, several studies from other groups have also shown that the mTORC1 pathway plays an important role in virus infection. It has been reported that HCMV infection activates the mTORC1 signaling in the infected cells[64]. EBV LMP1 upregulates Glut1 transcription to control aerobic glycolysis and tumorigenic growth of NPC cells through activation of mTORC1/NF-kappaB signaling[65]. Avian reovirus structural protein sigmaA activates the mTOC1/eIF4E/HIF-1α pathway to enhance glycolysis and the TCA cycle in the infected cells for virus replication[66]. In this study, we demonstrated that the phosphorylation of AMPK, a kinase that inhibits mTORC1 signaling[37, 38], was down-regulated following HHV-6A infection (**S4 Fig**), further supporting HHV-6 mediated-mTORC1 activation. In contrast, it has been reported that HCMV infection increases AMPK activity which was found to be necessary for glycolytic activation and high titer replication[67, 68]. These contradictory results suggest that the regulatory pathway and mechanism induced by HHV-6 infection might be different from other herpesvirus members.

Metabolism is a significant factor determining immune cells survival, proliferation, and the ability to perform its specific immune functions[69, 70]. Since viruses also rely on some similar nutrients and metabolic pathways used by immune cells, understanding the effects of modulating metabolic pathways by virus will be important in assessing the overall immune function of the host cells. Viruses may potentially take advantage of metabolism to alter immune responses to evade clearance[71]. HHV-6 is an important immunosuppressive virus and can utilize a variety of strategies to modulate or suppress host immune responses to facilitate virus spread and persistence [72, 73]. We speculate that manipulation of cell metabolism maybe another important strategy used by HHV-6 to escape antivirus immune responses and maintain the latency in host. Future studies should be aimed at identifying the interplay between metabolism and immune system during HHV-6 infection, which is critical to the development novel therapeutic strategies to restrain viral replication and enhance antiviral immune responses.

## Materials and methods

### Cells and viruses

The human T-lymphoblastoid cell line HSB-2 was purchased from the American Type Culture Collection (ATCC) and cultured in RPMI 1640 medium supplemented with 10% fetal bovine serum (FBS). The GS strain of HHV-6 variant A was propagated in cord blood mononuclear cells (CBMCs). Viral titers were determined as viral DNA equivalents by quantitative PCR. A multiplicity of infection (MOI) of 20 virus DNA copies per cell was used for all the experiments. The infection of HSB-2 cells by HHV-6A strain GS was performed as previously described[44].

### Chemical reagents

Rapamycin and Torin1 was purchased from the Selleck and used at 100 nM, and 5 nM respectively. 2-deoxy-D-glucose (2-DG) was also purchased from the Selleck and used at a final concentration of 1mM.

### Real-time quantitative RT- PCR analysis

Total RNA was extracted from mock and HHV-6A-infected HSB-2 cells using TRIzol reagent (Invitrogen, USA), cDNA was generated using a PrimeScript II RT kit (Takara) according to manufacturer’s instructions. Quantitative RT-PCR was performed on a 7500 fast PCR System (Applied Biosystems) using the TB Green Premix Ex Taq (Takara). Data were normalized to the expression of β-actin. Additionally, viral DNA was extracted from HHV-6A-infected T cells and supernatants, respectively, and viral DNA was quantified using the U22 primer set. Quantitative PCR primer pairs are listed in **S1 and S2 Tables**.

### Western-blot analysis

HHV-6A-infected and mock-infected cells were collected at 24, 48 and 72 h post-infection. Cells were lysed in cell lysis buffer at 4°C for 30 min. Protein concentrations of the cell lysates were measured using the BCA reagent (Beyotime Biotechnology). Equivalent amounts of proteins were subjected to sodium dodecyl sulfate-polyacrylamide gel electrophoresis (SDS-PAGE). Proteins were transferred to polyvinylidene difluoride (PVDF) membranes (BIO-RAD) and detected with the corresponding primary and secondary antibodies. The protein bands were visualized using the enhanced chemiluminescence (ECL) system (Tanon Science & Technology). The antibodies used in Western-blotting analyses included anti–mTOR, anti–phospho-mTOR, anti-p70S6K, anti–phospho-p70S6K, anti-4E-BP1, anti–phospho-4E-BP1, anti-AKT, anti-phospho-AKT, anti-TSC2, anti-phospho-TSC2 (Cell Signaling Technology), anti-β-actin, anti-Glut1 and anti-Glut3 (Proteintech Biotechnology). A monoclonal antibody against the HHV-6 IE1 and gB was produced by our laboratory.

### Immunofluorescence

HHV-6A-infected and mock-infected cells were collected at 72 h post-infection. The cells were fixed with acetone/methanol mixture, and stained with anti-Glut1 or anti-Glut3 primary antibodies (Proteintech Biotechnology) followed by FITC-labeled goat anti-Rabbit IgG (H+L). Cells were mounted in medium containing DAPI (4’,6’-diamidino-2-phenylindole) before they were observed under an Olympus BX53 fluorescence microscope (Olympus, Inc.).

### Glucose uptake assay

HSB-2 cells were infected with or without HHV-6A for the indicated time points. The 1640 culture medium was replaced by glucose-free medium with 10% FBS. After incubating the cells for another 30 min, 2-NBDG (Cayman Chemical, USA) was added at a final concentration of 100 μM for 15 min and cells were collected and washed twice with PBS. The cells were ready to be analyzed by flow cytometry.

### Measurements of glucose consumption and lactate production

Cells (5× 10^5^/well) were seeded in a 6-well plate, and infected with HHV-6A in the presence or absence of inhibitors (Rapamycin or 2-DG). Mock-infected and HHV-6A-infected HSB-2 cells were cultured for 24, 48 and 72 h. Glucose and lactate concentrations in the culture media were measured using the Glucose Oxidation Assay Kit (Applygen Technologies) and the Lactate Assay Kit (Jiancheng Technologies), respectively, according to the manufacturer’s instructions. Glucose consumption and lactate measurements were normalized to cell numbers.

### Metabolic assays

The measurement of extracellular acidification rate (ECAR) were determined with the Seahorse XF96 Extracellular Flux Analyzer (Seahorse Bioscience). Experiments were performed according to the manufacturer's protocols. Briefly, mock and HHV-6A-infected HSB-2 cells were cultured for 24 h, and the cells were then plated on cell-tak-coated Seahorse XF96 cell-culture microplates at a density of 5×10^4^ cells per well. The ECAR were measured under basal condition after sequentially adding 10mM glucose, 4μM oligomycin and 100mM 2-DG (XF Glycolysis Stress Test kit, Agilent, USA) at indicated time points. For some experiments, the metabolic profiles were evaluated in the presence or absence of rapamycin in cultured HSB-2 cells. The parameters of cellular glycolysis (including glycolysis, glycolytic capacity and glycolytic reserve) were calculated from ECAR profiles. Glycolysis was determined after the addition of glucose, and the glycolytic capacity was assessed after the addition of oligomycin. Glycolytic reserve was defined as the difference between glycolytic capacity and glycolysis.

### RNA sequencing analysis

Mock-infected and HHV-6A-infected HSB-2 cells were cultured for 72 h. Total RNA was extracted using TRIzol reagent (Invitrogen, USA) following the manufacturer’s instructions. A total amount of 3 μg of RNA per sample was used as input material for the RNA sample preparations. Sequencing libraries were generated using NEBNext Ultra Directional RNA Library Prep Kit for Illumina (NEB, USA) following the manufacturer’s recommendations. After cluster generation, the library preparations were sequenced on an Illumina Hiseq 2500 platform by CapitalBio Technology. Differentially expressed genes were identified by fold-change screening. Gene ontology (GO) analysis and Kyoto Encyclopedia of Genes and Genomes (KEGG) pathway enrichment analysis were performed to systematically identify differentially expressed genes and the pathways associated with HHV-6A infection.

### Viral genome and protein quantification

Cells (5 × 10^5^/well) were infected with HHV-6A and seeded in a 6-well plate with or without inhibitors (2-DG, Rapamycin or Torin). The medium was replaced with fresh medium at 48 h post-infection, and the cells were incubated for an additional 24 h. Total DNA was extracted from HHV-6A-infeted cells and supernatant respectively. Viral genomes were quantified by the real-time PCR using the primers for viral U22 gene detection. HHV-6 immediate early protein, IE1 and late protein, gB were detected by Western blot analysis.

### Statistical analysis

Statistical analysis was performed using GraphPad Prism5 software. The data are presented as mean ± standard deviation (SD). Statistical significance was determined by a Student’s t-test or one-way analysis of variance (ANOVA). P value of < 0.05 was considered significant.

## Supporting information

S1 Fig Confirmation of HHV-6 infection in HSB-2 cells. **(A)** HHV-6A infection exhibited typical cytopathic effects in infected HSB-2 cells. The morphological characteristics of mock-infected or HHV-6A-infected HSB-2 cells were observed under a light microscope at various time points postinfection. Scale bar, 50 μm. **(B)** HHV-6 gB expression on mock-infected or HHV-6A-infected HSB-2 cells was determined by immunofluorescence analysis. Scale bar, 20 μm. Mock- and HHV-6A-infected HSB-2 cells were stained for gB (green) and DNA (blue) with an anti-gB antibody and DAPI stain at 72 h postinfection. The percentages of cells positive for HHV-6 gB are shown in the histograms on the right. (C) HHV-6 gB expression on mock-infected or HHV-6A-infected HSB-2 cells was determined by Western blot analysis with anti-gB antibody.
(TIF)

S2 Fig Gene expression levels of Glut family in HSB-2 cells. The total RNA in HSB-2 cells was isolated and then mRNA levels were analyzed by quantitative RT-PCR. The expression levels of each gene were normalized to β-actin expression levels and adjust to the levels in Glut1 (served as 1). Data shown are mean ± SD from three independent experiments. N.D. = not detected.
(TIF)

S3 Fig HHV-6 infection significantly up-regulated mRNA levels of key TCA cycle enzymes in HSB-2 cells. HSB-2 cells were mock infected or infected with HHV-6A. The total RNA was isolated at 24, 48, and 72 hpi and then mRNA levels were analyzed by quantitative PCR. The expression levels of each gene were normalized to β-actin and plotted with respect to mock infection. Data shown are mean ± SD from three independent experiments.
(TIF)

S4 Fig HHV-6A infection down-regulates the AMPK expression. Mock infected and HHV-6A infected cells were lysed and analyzed by Western blotting using specific antibodies against AMPK and phosphorylated AMPK. Phosphorylated AMPK protein levels were quantitatively analyzed and were compared with β-actin expression with a densitometer. Results are means ± SD from three independent experiments. * p<0.05, **p<0.01, compared with the mock-infected group.
(TIF)

S5 Fig 2-DG blocks HHV-6-mediated glycolytic activation. HSB-2 cells were mock infected or infected with HHV-6A. After adsorption, cells were treated with the glycolysis inhibitor 2-DG (1 mM) or DMSO. **(A)** 2-DG treatment significantly decreased glucose uptake in HHV-6-infected cells. Glucose uptake was determined by flow cytometry with addition of 2-NBDG for 15 min after 72 h culture. **(B)** 2-DG treatment increased glucose levels in the culture medium of HHV-6A infected HSB-2 cells. The glucose levels in the culture medium were determined after 72 h culture using a Glucose Oxidation Assay Kit. Results shown in histogram are mean ± SD from three independent experiments. * p<0.05, ** p<0.01, compared with the indicated control group. **(C)** 2-DG treatment decreased lactate secretion of HSB-2 cell. The lactate levels in culture supernatant was analyzed at 72 h post infection. Results shown in the histogram are mean ± SD from three independent experiments. ** p<0.01, compared with the indicated control group.
(TIF)

S1 Table Primers used for real-time quantitative RT- PCR (Glycolytic enzymes). (DOCX)

S2 Table Primers used for quantitative PCR (HHV-6 U22). (DOCX)

S1 Data The numerical data and statistical analysis that were used to generate graphs in the manuscript. (XLSX)

## References

[1] De Bolle L, Naesens L, De Clercq E. Update on human herpesvirus 6 biology, clinical features, and therapy. Clinical microbiology reviews. 2005;18(1):217–45. Epub 2005/01/18. 10.1128/CMR.18.1.217-245.2005 15653828 PMC544175

[2] Agut H, Bonnafous P, Gautheret-Dejean A. Laboratory and clinical aspects of human herpesvirus 6 infections. Clinical microbiology reviews. 2015;28(2):313–35. Epub 2015/03/13. 10.1128/CMR.00122-14 25762531 PMC4402955

[3] Pantry SN, Medveczky PG. Latency, Integration, and Reactivation of Human Herpesvirus-6. Viruses. 2017;9(7). Epub 2017/07/25. 10.3390/v9070194 28737715 PMC5537686

[4] Pellett PE, Ablashi DV, Ambros PF, Agut H, Caserta MT, Descamps V, et al Chromosomally integrated human herpesvirus 6: questions and answers. Reviews in medical virology. 2012;22(3):144–55. Epub 2011/11/05. 10.1002/rmv.715 22052666 PMC3498727

[5] Ablashi D, Agut H, Alvarez-Lafuente R, Clark DA, Dewhurst S, DiLuca D, et al Classification of HHV-6A and HHV-6B as distinct viruses. Archives of virology. 2014;159(5):863–70. Epub 2013/11/07. 10.1007/s00705-013-1902-5 24193951 PMC4750402

[6] Caselli E, Di Luca D. Molecular biology and clinical associations of Roseoloviruses human herpesvirus 6 and human herpesvirus 7. The new microbiologica. 2007;30(3):173–87. Epub 2007/09/07. . 17802896

[7] Yamanishi K, Okuno T, Shiraki K, Takahashi M, Kondo T, Asano Y, et al Identification of human herpesvirus-6 as a causal agent for exanthem 10.1016/s0140-6736(88)91893-4 Lancet. 1988;1(8594):1065–7. Epub 1988/05/14. 2896909

[8] Zerr DM, Boeckh M, Delaney C, Martin PJ, Xie H, Adler AL, et al HHV-6 reactivation and associated sequelae after hematopoietic cell transplantation. Biology of blood and marrow transplantation: journal of the American Society for Blood and Marrow Transplantation. 2012;18(11):1700–8. Epub 2012/05/30. 10.1016/j.bbmt.2012.05.012 22641196 PMC3439599

[9] Aoki J, Numata A, Yamamoto E, Fujii E, Tanaka M, Kanamori H. Impact of Human Herpesvirus-6 Reactivation on Outcomes of Allogeneic Hematopoietic Stem Cell Transplantation. Biology of blood and marrow transplantation: journal of the American Society for Blood and Marrow Transplantation. 2015;21(11):2017–22. Epub 2015/08/01. 10.1016/j.bbmt.2015.07.022 . 26226409

[10] Leibovitch EC, Jacobson S. Evidence linking HHV-6 with multiple sclerosis: an update. Current opinion in virology. 2014;9:127–33. Epub 2014/12/03. 10.1016/j.coviro.2014.09.016 25462444 PMC4269240

[11] Thomas D, Liakos V, Michou V, Kapranos N, Kaltsas G, Tsilivakos V, et al Detection of herpes virus DNA in post-operative thyroid tissue specimens of patients with autoimmune thyroid disease. Experimental and clinical endocrinology & diabetes: official journal, German Society of Endocrinology [and] German Diabetes Association. 2008;116(1):35–9. Epub 2008/02/02. 10.1055/s-2007-956171 . 18240111

[12] Caselli E, Zatelli MC, Rizzo R, Benedetti S, Martorelli D, Trasforini G, et al Virologic and immunologic evidence supporting an association between HHV-6 and Hashimoto's thyroiditis. PLoS pathogens. 2012;8(10):e1002951 Epub 2012/10/12. 10.1371/journal.ppat.1002951 23055929 PMC3464215

[13] Readhead B, Haure-Mirande JV, Funk CC, Richards MA, Shannon P, Haroutunian V, et al Multiscale Analysis of Independent Alzheimer's Cohorts Finds Disruption of Molecular, Genetic, and Clinical Networks by Human Herpesvirus. Neuron. 2018;99(1):64–82 e7. Epub 2018/06/26. 10.1016/j.neuron.2018.05.023 . 29937276 PMC6551233

[14] Chi J, Gu B, Zhang C, Peng G, Zhou F, Chen Y, et al Human herpesvirus 6 latent infection in patients with glioma. The Journal of infectious diseases. 2012;206(9):1394–8. Epub 2012/09/11. 10.1093/infdis/jis513 . 22962688

[15] Goodwin CM, Xu S, Munger J. Stealing the Keys to the Kitchen: Viral Manipulation of the Host Cell Metabolic Network. Trends in microbiology. 2015;23(12):789–98. Epub 2015/10/07. 10.1016/j.tim.2015.08.007 26439298 PMC4679435

[16] Mushtaq M, Darekar S, Kashuba E. DNA Tumor Viruses and Cell Metabolism. Oxidative medicine and cellular longevity. 2016;2016:6468342 Epub 2016/04/02. 10.1155/2016/6468342 27034740 PMC4789518

[17] Munger J, Bajad SU, Coller HA, Shenk T, Rabinowitz JD. Dynamics of the cellular metabolome during human cytomegalovirus infection. PLoS pathogens. 2006;2(12):e132 Epub 2006/12/19. 10.1371/journal.ppat.0020132 17173481 PMC1698944

[18] Chambers JW, Maguire TG, Alwine JC. Glutamine metabolism is essential for human cytomegalovirus infection. Journal of virology. 2010;84(4):1867–73. Epub 2009/11/27. 10.1128/JVI.02123-09 19939921 PMC2812398

[19] Spencer CM, Schafer XL, Moorman NJ, Munger J. Human cytomegalovirus induces the activity and expression of acetyl-coenzyme A carboxylase, a fatty acid biosynthetic enzyme whose inhibition attenuates viral replication. Journal of virology. 2011;85(12):5814–24. Epub 2011/04/08. 10.1128/JVI.02630-10 21471234 PMC3126312

[20] Yu Y, Maguire TG, Alwine JC. Human cytomegalovirus activates glucose transporter 4 expression to increase glucose uptake during infection. Journal of virology. 2011;85(4):1573–80. Epub 2010/12/15. 10.1128/JVI.01967-10 21147915 PMC3028904

[21] Delgado T, Carroll PA, Punjabi AS, Margineantu D, Hockenbery DM, Lagunoff M. Induction of the Warburg effect by Kaposi's sarcoma herpesvirus is required for the maintenance of latently infected endothelial cells. Proceedings of the National Academy of Sciences of the United States of America. 2010;107(23):10696–701. Epub 2010/05/26. 10.1073/pnas.1004882107 20498071 PMC2890792

[22] Delgado T, Sanchez EL, Camarda R, Lagunoff M. Global metabolic profiling of infection by an oncogenic virus: KSHV induces and requires lipogenesis for survival of latent infection. PLoS pathogens. 2012;8(8):e1002866 Epub 2012/08/24. 10.1371/journal.ppat.1002866 22916018 PMC3420960

[23] Xiao L, Hu ZY, Dong X, Tan Z, Li W, Tang M, et al Targeting Epstein-Barr virus oncoprotein LMP1-mediated glycolysis sensitizes nasopharyngeal carcinoma to radiation therapy. Oncogene. 2014;33(37):4568–78. Epub 2014/03/26. 10.1038/onc.2014.32 24662831 PMC4162460

[24] Vastag L, Koyuncu E, Grady SL, Shenk TE, Rabinowitz JD. Divergent effects of human cytomegalovirus and herpes simplex virus-1 on cellular metabolism. PLoS pathogens. 2011;7(7):e1002124 Epub 2011/07/23. 10.1371/journal.ppat.1002124 21779165 PMC3136460

[25] Jacobs SR, Herman CE, Maciver NJ, Wofford JA, Wieman HL, Hammen JJ, et al Glucose uptake is limiting in T cell activation and requires CD28-mediated Akt-dependent and independent pathways. J Immunol. 2008;180(7):4476–86. 10.4049/jimmunol.180.7.4476 18354169 PMC2593791

[26] Wofford JA, Wieman HL, Jacobs SR, Zhao Y, Rathmell JC. IL-7 promotes Glut1 trafficking and glucose uptake via STAT5-mediated activation of Akt to support T-cell survival. Blood. 2008;111(4):2101–11. Epub 2007/11/29. 10.1182/blood-2007-06-096297 18042802 PMC2234050

[27] Loisel-Meyer S, Swainson L, Craveiro M, Oburoglu L, Mongellaz C, Costa C, et al Glut1-mediated glucose transport regulates HIV infection. Proceedings of the National Academy of Sciences of the United States of America. 2012;109(7):2549–54. Epub 2012/02/07. 10.1073/pnas.1121427109 22308487 PMC3289356

[28] Gonnella R, Santarelli R, Farina A, Granato M, D'Orazi G, Faggioni A, et al Kaposi sarcoma associated herpesvirus (KSHV) induces AKT hyperphosphorylation, bortezomib-resistance and GLUT-1 plasma membrane exposure in THP-1 monocytic cell line. Journal of experimental & clinical cancer research: CR. 2013;32:79 Epub 2014/01/16. 10.1186/1756-9966-32-79 24422998 PMC3874756

[29] Sommermann TG, O'Neill K, Plas DR, Cahir-McFarland E. IKKbeta and NF-kappaB transcription govern lymphoma cell survival through AKT-induced plasma membrane trafficking of GLUT1. Cancer research. 2011;71(23):7291–300. Epub 2011/10/12. 10.1158/0008-5472.CAN-11-1715 21987722 PMC3228879

[30] Dukhande VV, Sharma GC, Lai JC, Farahani R. Chronic hypoxia-induced alterations of key enzymes of glucose oxidative metabolism in developing mouse liver are mTOR dependent. Molecular and cellular biochemistry. 2011;357(1–2):189–97. Epub 2011/06/01. 10.1007/s11010-011-0889-z . 21625955

[31] Mori H, Inoki K, Munzberg H, Opland D, Faouzi M, Villanueva EC, et al Critical role for hypothalamic mTOR activity in energy balance. Cell metabolism. 2009;9(4):362–74. Epub 2009/04/10. 10.1016/j.cmet.2009.03.005 19356717 PMC2790375

[32] Sekulic A, Hudson CC, Homme JL, Yin P, Otterness DM, Karnitz LM, et al A direct linkage between the phosphoinositide 3-kinase-AKT signaling pathway and the mammalian target of rapamycin in mitogen-stimulated and transformed cells. Cancer research. 2000;60(13):3504–13. Epub 2000/07/26. . 10910062

[33] Chen J, Hu CF, Hou JH, Shao Q, Yan LX, Zhu XF, et al Epstein-Barr virus encoded latent membrane protein 1 regulates mTOR signaling pathway genes which predict poor prognosis of nasopharyngeal carcinoma. Journal of translational medicine. 2010;8:30 Epub 2010/03/27. 10.1186/1479-5876-8-30 20338061 PMC2861642

[34] Johnson RA, Wang X, Ma XL, Huong SM, Huang ES. Human cytomegalovirus up-regulates the phosphatidylinositol 3-kinase (PI3-K) pathway: inhibition of PI3-K activity inhibits viral replication and virus-induced signaling. Journal of virology. 2001;75(13):6022–32. Epub 2001/06/08. 10.1128/JVI.75.13.6022-6032.2001 11390604 PMC114318

[35] Saxton RA, Sabatini DM. mTOR Signaling in Growth, Metabolism, and Disease. Cell. 2017;169(2):361–71. Epub 2017/04/08. 10.1016/j.cell.2017.03.035 . 28388417

[36] Dan HC, Sun M, Yang L, Feldman RI, Sui XM, Ou CC, et al Phosphatidylinositol 3-kinase/Akt pathway regulates tuberous sclerosis tumor suppressor complex by phosphorylation of tuberin. The Journal of biological chemistry. 2002;277(38):35364–70. Epub 2002/08/09. 10.1074/jbc.M205838200 . 12167664

[37] Shaw RJ. LKB1 and AMP-activated protein kinase control of mTOR signalling and growth. Acta Physiol (Oxf). 2009;196(1):65–80. Epub 2009/02/28. 10.1111/j.1748-1716.2009.01972.x 19245654 PMC2760308

[38] Garcia D, Shaw RJ. AMPK: Mechanisms of Cellular Energy Sensing and Restoration of Metabolic Balance. Molecular cell. 2017;66(6):789–800. Epub 2017/06/18. 10.1016/j.molcel.2017.05.032 28622524 PMC5553560

[39] Salahuddin SZ, Ablashi DV, Markham PD, Josephs SF, Sturzenegger S, Kaplan M, et al Isolation of a new virus, HBLV, in patients with lymphoproliferative disorders. Science. 1986;234(4776):596–601. Epub 1986/10/31. 10.1126/science.2876520 . 2876520

[40] Takahashi K, Sonoda S, Higashi K, Kondo T, Takahashi H, Takahashi M, et al Predominant CD4 T-lymphocyte tropism of human herpesvirus 6-related virus. Journal of virology. 1989;63(7):3161–3. Epub 1989/07/01. 2542623 10.1128/jvi.63.7.3161-3163.1989 PMC250875

[41] Yasukawa M, Inoue Y, Ohminami H, Terada K, Fujita S. Apoptosis of CD4+ T lymphocytes in human herpesvirus-6 infection. The Journal of general virology. 1998;79 (Pt 1):143–7. Epub 1998/02/14. 10.1099/0022-1317-79-1-143 . 9460935

[42] Gupta S, Agrawal S, Gollapudi S. Differential effect of human herpesvirus 6A on cell division and apoptosis among naive and central and effector memory CD4+ and CD8+ T-cell subsets. Journal of virology. 2009;83(11):5442–50. Epub 2009/03/20. 10.1128/JVI.00106-09 19297473 PMC2681935

[43] Iampietro M, Morissette G, Gravel A, Flamand L. Inhibition of interleukin-2 gene expression by human herpesvirus 6B U54 tegument protein. Journal of virology. 2014;88(21):12452–63. Epub 2014/08/15. 10.1128/JVI.02030-14 25122797 PMC4248915

[44] Li L, Gu B, Zhou F, Chi J, Wang F, Peng G, et al Human herpesvirus 6 suppresses T cell proliferation through induction of cell cycle arrest in infected cells in the G2/M phase. Journal of virology. 2011;85(13):6774–83. Epub 2011/04/29. 10.1128/JVI.02577-10 21525341 PMC3126536

[45] Ota M, Serada S, Naka T, Mori Y. MHC class I molecules are incorporated into human herpesvirus-6 viral particles and released into the extracellular environment. Microbiology and immunology. 2014;58(2):119–25. Epub 2013/12/18. 10.1111/1348-0421.12121 . 24330265

[46] Sullivan BM, Coscoy L. Downregulation of the T-cell receptor complex and impairment of T-cell activation by human herpesvirus 6 u24 protein. Journal of virology. 2008;82(2):602–8. Epub 2007/11/06. 10.1128/JVI.01571-07 17977973 PMC2224597

[47] Mayer KA, Stockl J, Zlabinger GJ, Gualdoni GA. Hijacking the Supplies: Metabolism as a Novel Facet of Virus-Host Interaction. Frontiers in immunology. 2019;10:1533 10.3389/fimmu.2019.01533 31333664 PMC6617997

[48] Thaker SK, Ch'ng J, Christofk HR. Viral hijacking of cellular metabolism. BMC Biol. 2019;17(1):59 10.1186/s12915-019-0678-9 31319842 PMC6637495

[49] Sanchez EL, Lagunoff M. Viral activation of cellular metabolism. Virology. 2015;479–480:609–18. Epub 2015/03/31. 10.1016/j.virol.2015.02.038 25812764 PMC4424078

[50] Abrantes JL, Alves CM, Costa J, Almeida FC, Sola-Penna M, Fontes CF, et al Herpes simplex type 1 activates glycolysis through engagement of the enzyme 6-phosphofructo-1-kinase (PFK-1). Biochimica et biophysica acta. 2012;1822(8):1198–206. Epub 2012/05/01. 10.1016/j.bbadis.2012.04.011 . 22542512

[51] Lo AK, Dawson CW, Young LS, Ko CW, Hau PM, Lo KW. Activation of the FGFR1 signalling pathway by the Epstein-Barr virus-encoded LMP1 promotes aerobic glycolysis and transformation of human nasopharyngeal epithelial cells. The Journal of pathology. 2015;237(2):238–48. Epub 2015/06/23. 10.1002/path.4575 . 26096068

[52] Yogev O, Lagos D, Enver T, Boshoff C. Kaposi's sarcoma herpesvirus microRNAs induce metabolic transformation of infected cells. PLoS pathogens. 2014;10(9):e1004400 Epub 2014/09/26. 10.1371/journal.ppat.1004400 25255370 PMC4177984

[53] Thai M, Graham NA, Braas D, Nehil M, Komisopoulou E, Kurdistani SK, et al Adenovirus E4ORF1-induced MYC activation promotes host cell anabolic glucose metabolism and virus replication. Cell metabolism. 2014;19(4):694–701. Epub 2014/04/08. 10.1016/j.cmet.2014.03.009 24703700 PMC4294542

[54] Fontaine KA, Sanchez EL, Camarda R, Lagunoff M. Dengue virus induces and requires glycolysis for optimal replication. Journal of virology. 2015;89(4):2358–66. Epub 2014/12/17. 10.1128/JVI.02309-14 25505078 PMC4338897

[55] Mueckler M, Thorens B. The SLC2 (GLUT) family of membrane transporters. Mol Aspects Med. 2013;34(2–3):121–38. 10.1016/j.mam.2012.07.001 23506862 PMC4104978

[56] Li L, Liu X, Sanders KL, Edwards JL, Ye J, Si F, et al TLR8-Mediated Metabolic Control of Human Treg Function: A Mechanistic Target for Cancer Immunotherapy. Cell metabolism. 2019;29(1):103–23 e5. Epub 2018/10/23. 10.1016/j.cmet.2018.09.020 . 30344014 PMC7050437

[57] Fu Y, Maianu L, Melbert BR, Garvey WT. Facilitative glucose transporter gene expression in human lymphocytes, monocytes, and macrophages: a role for GLUT isoforms 1, 3, and 5 in the immune response and foam cell formation. Blood Cells Mol Dis. 2004;32(1):182–90. 10.1016/j.bcmd.2003.09.002 . 14757434

[58] Simpson IA, Dwyer D, Malide D, Moley KH, Travis A, Vannucci SJ. The facilitative glucose transporter GLUT3: 20 years of distinction. Am J Physiol Endocrinol Metab. 2008;295(2):E242–53. 10.1152/ajpendo.90388.2008 18577699 PMC2519757

[59] Roberts DJ, Miyamoto S. Hexokinase II integrates energy metabolism and cellular protection: Akting on mitochondria and TORCing to autophagy. Cell death and differentiation. 2015;22(2):248–57. Epub 2014/10/18. 10.1038/cdd.2014.173 25323588 PMC4291497

[60] Al Hasawi N, Alkandari MF, Luqmani YA. Phosphofructokinase: a mediator of glycolytic flux in cancer progression. Critical reviews in oncology/hematology. 2014;92(3):312–21. Epub 2014/06/10. 10.1016/j.critrevonc.2014.05.007 . 24910089

[61] Xu D, Liang J, Lin J, Yu C. PKM2: A Potential Regulator of Rheumatoid Arthritis via Glycolytic and Non-Glycolytic Pathways. Frontiers in immunology. 2019;10:2919 Epub 2020/01/11. 10.3389/fimmu.2019.02919 31921178 PMC6930793

[62] Duvel K, Yecies JL, Menon S, Raman P, Lipovsky AI, Souza AL, et al Activation of a metabolic gene regulatory network downstream of mTOR complex 1. Molecular cell. 2010;39(2):171–83. Epub 2010/07/31. 10.1016/j.molcel.2010.06.022 20670887 PMC2946786

[63] Liu C, Chapman NM, Karmaus PW, Zeng H, Chi H. mTOR and metabolic regulation of conventional and regulatory T cells. Journal of leukocyte biology. 2015;97(5):837–47. Epub 2015/02/26. 10.1189/jlb.2RI0814-408R 25714803 PMC4398256

[64] Moorman NJ, Shenk T. Rapamycin-resistant mTORC1 kinase activity is required for herpesvirus replication. Journal of virology. 2010;84(10):5260–9. Epub 2010/02/26. 10.1128/JVI.02733-09 20181700 PMC2863801

[65] Zhang J, Jia L, Lin W, Yip YL, Lo KW, Lau VM, et al Epstein-Barr Virus-Encoded Latent Membrane Protein 1 Upregulates Glucose Transporter 1 Transcription via the mTORC1/NF-kappaB Signaling Pathways. Journal of virology. 2017;91(6). Epub 2017/01/06. 10.1128/JVI.02168-16 28053105 PMC5331802

[66] Chi PI, Huang WR, Chiu HC, Li JY, Nielsen BL, Liu HJ. Avian reovirus sigmaA-modulated suppression of lactate dehydrogenase and upregulation of glutaminolysis and the mTOC1/eIF4E/HIF-1alpha pathway to enhance glycolysis and the TCA cycle for virus replication. Cellular microbiology. 2018:e12946 Epub 2018/08/30. 10.1111/cmi.12946 . 30156372

[67] Terry LJ, Vastag L, Rabinowitz JD, Shenk T. Human kinome profiling identifies a requirement for AMP-activated protein kinase during human cytomegalovirus infection. Proceedings of the National Academy of Sciences of the United States of America. 2012;109(8):3071–6. Epub 2012/02/09. 10.1073/pnas.1200494109 22315427 PMC3286917

[68] McArdle J, Moorman NJ, Munger J. HCMV targets the metabolic stress response through activation of AMPK whose activity is important for viral replication. PLoS pathogens. 2012;8(1):e1002502 Epub 2012/02/01. 10.1371/journal.ppat.1002502 22291597 PMC3266935

[69] Buck MD, Sowell RT, Kaech SM, Pearce EL. Metabolic Instruction of Immunity. Cell. 2017;169(4):570–86. 10.1016/j.cell.2017.04.004 28475890 PMC5648021

[70] McKinney EF, Smith KGC. Metabolic exhaustion in infection, cancer and autoimmunity. Nature immunology. 2018;19(3):213–21. 10.1038/s41590-018-0045-y . 29403049

[71] Moreno-Altamirano MMB, Kolstoe SE, Sanchez-Garcia FJ. Virus Control of Cell Metabolism for Replication and Evasion of Host Immune Responses. Front Cell Infect Microbiol. 2019;9:95 Epub 2019/05/07. 10.3389/fcimb.2019.00095 31058096 PMC6482253

[72] Schmiedel D, Tai J, Levi-Schaffer F, Dovrat S, Mandelboim O. Human Herpesvirus 6B Downregulates Expression of Activating Ligands during Lytic Infection To Escape Elimination by Natural Killer Cells. Journal of virology. 2016;90(21):9608–17. Epub 2016/08/19. 10.1128/JVI.01164-16 27535049 PMC5068514

[73] Dagna L, Pritchett JC, Lusso P. Immunomodulation and immunosuppression by human herpesvirus 6A and 6 B. Future virology. 2013;8(3):273–87. Epub 2013/10/29. 10.2217/fvl.13.7 24163703 PMC3806647

