# Supplementary figures and images for: Human herpesvirus 6A promotes glycolysis in infected T cells by activation of mTOR signaling
Source: PLoS Pathog. 2020 Jun 9;16(6):e1008568. doi: 10.1371/journal.ppat.1008568 (PMC7282626; doi:10.1371/journal.ppat.1008568)

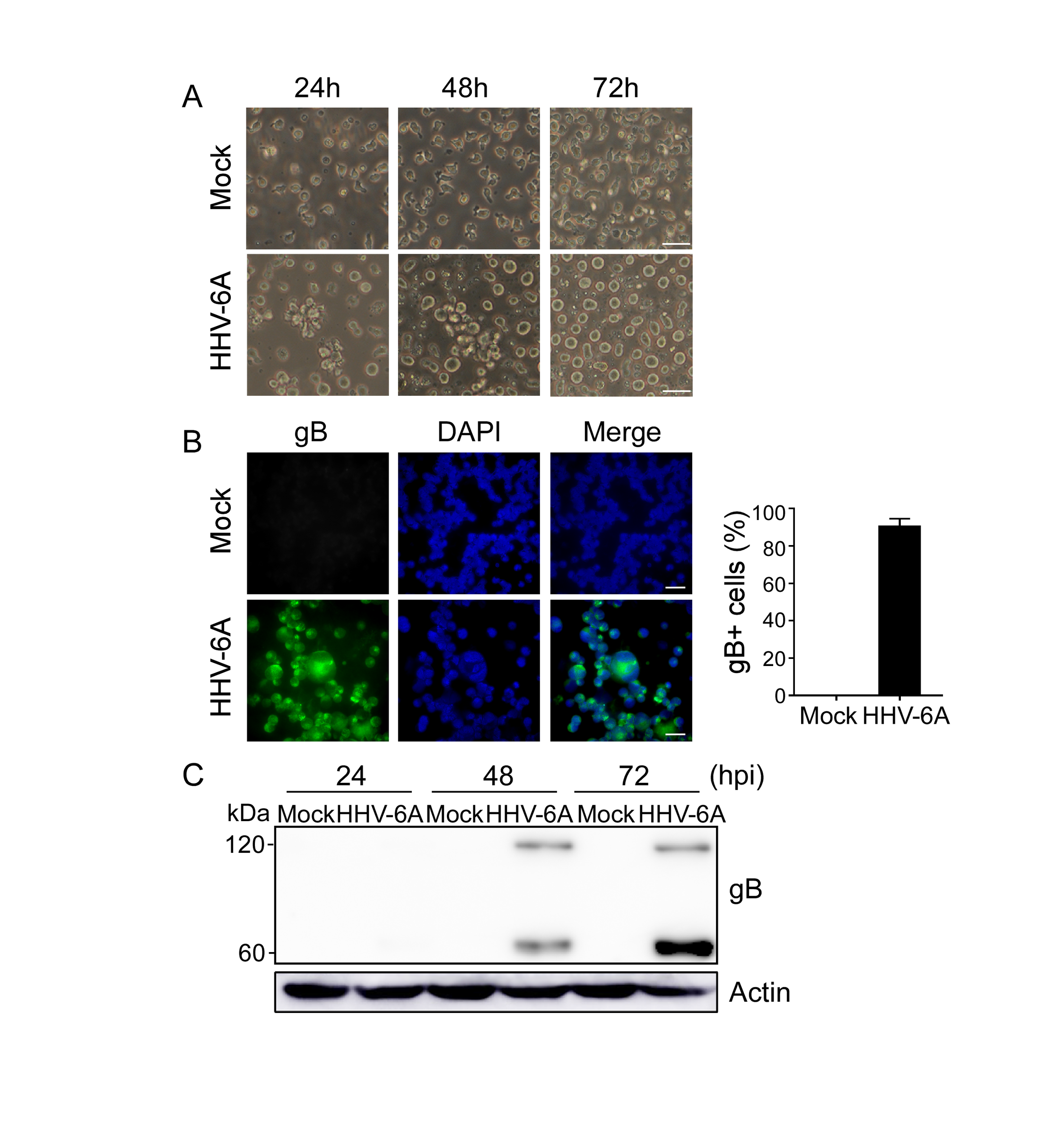

Supplement: S1 Fig — (A) HHV-6A infection exhibited typical cytopathic effects in infected HSB-2 cells. The morphological characteristics of mock-infected or HHV-6A-infected HSB-2 cells were observed under a light microscope at various time points postinfection. Scale bar, 50 μm. (B) HHV-6 gB expression on mock-infected or HHV-6A-infected HSB-2 cells was determined by immunofluorescence analysis. Scale bar, 20 μm. Mock- and HHV-6A-infected HSB-2 cells were stained for gB (green) and DNA (blue) with an anti-gB antibody and DAPI stain at 72 h postinfection. The percentages of cells positive for HHV-6 gB are shown in the histograms on the right. (C) HHV-6 gB expression on mock-infected or HHV-6A-infected HSB-2 cells was determined by Western blot analysis with anti-gB antibody. (TIF) [file ppat.1008568.s001.tif]

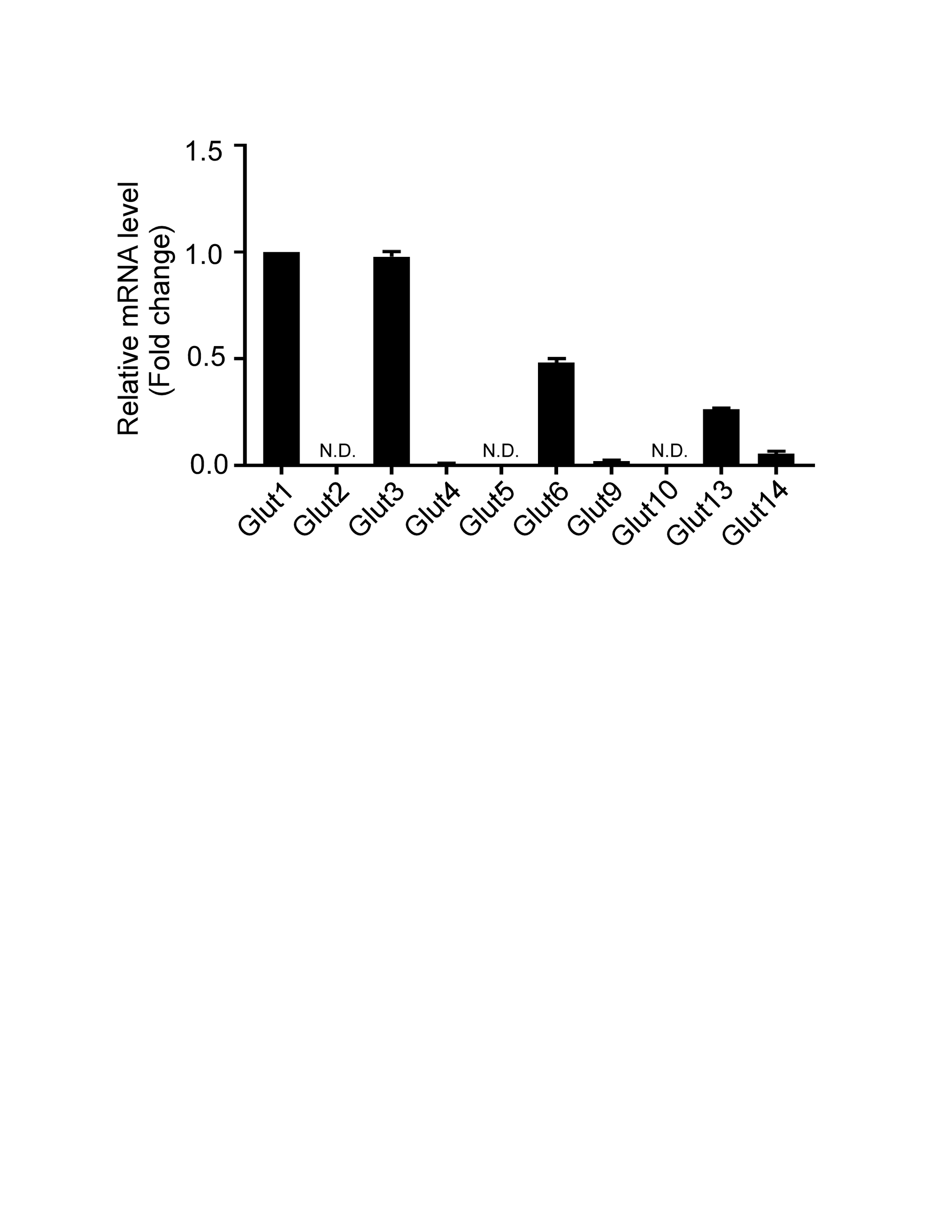

Supplement: S2 Fig — The total RNA in HSB-2 cells was isolated and then mRNA levels were analyzed by quantitative RT-PCR. The expression levels of each gene were normalized to β-actin expression levels and adjust to the levels in Glut1 (served as 1). Data shown are mean ± SD from three independent experiments. N.D. = not detected. (TIF) [file ppat.1008568.s002.tif]

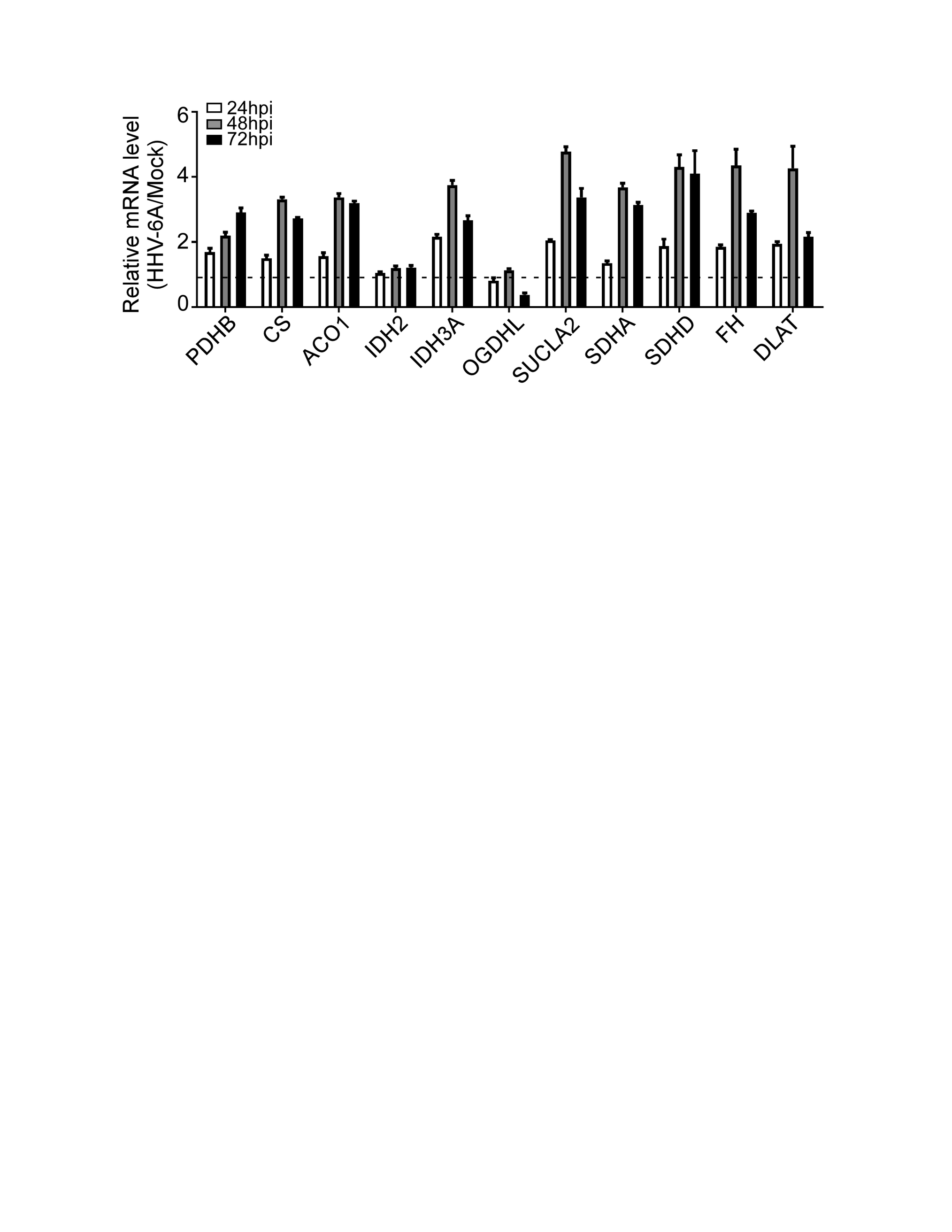

Supplement: S3 Fig — HSB-2 cells were mock infected or infected with HHV-6A. The total RNA was isolated at 24, 48, and 72 hpi and then mRNA levels were analyzed by quantitative PCR. The expression levels of each gene were normalized to β-actin and plotted with respect to mock infection. Data shown are mean ± SD from three independent experiments. (TIF) [file ppat.1008568.s003.tif]

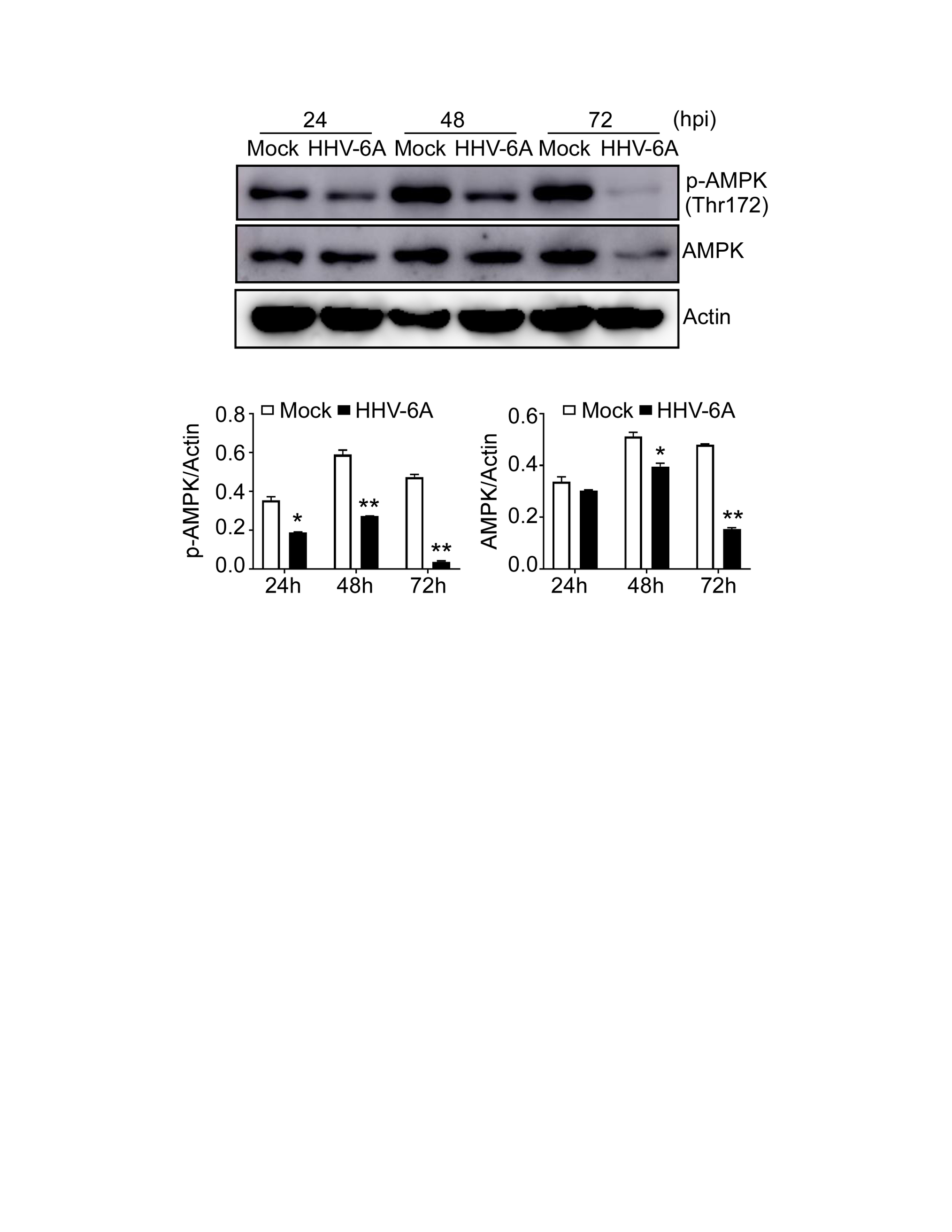

Supplement: S4 Fig — Mock infected and HHV-6A infected cells were lysed and analyzed by Western blotting using specific antibodies against AMPK and phosphorylated AMPK. Phosphorylated AMPK protein levels were quantitatively analyzed and were compared with β-actin expression with a densitometer. Results are means ± SD from three independent experiments. * p<0.05, **p<0.01, compared with the mock-infected group. (TIF) [file ppat.1008568.s004.tif]

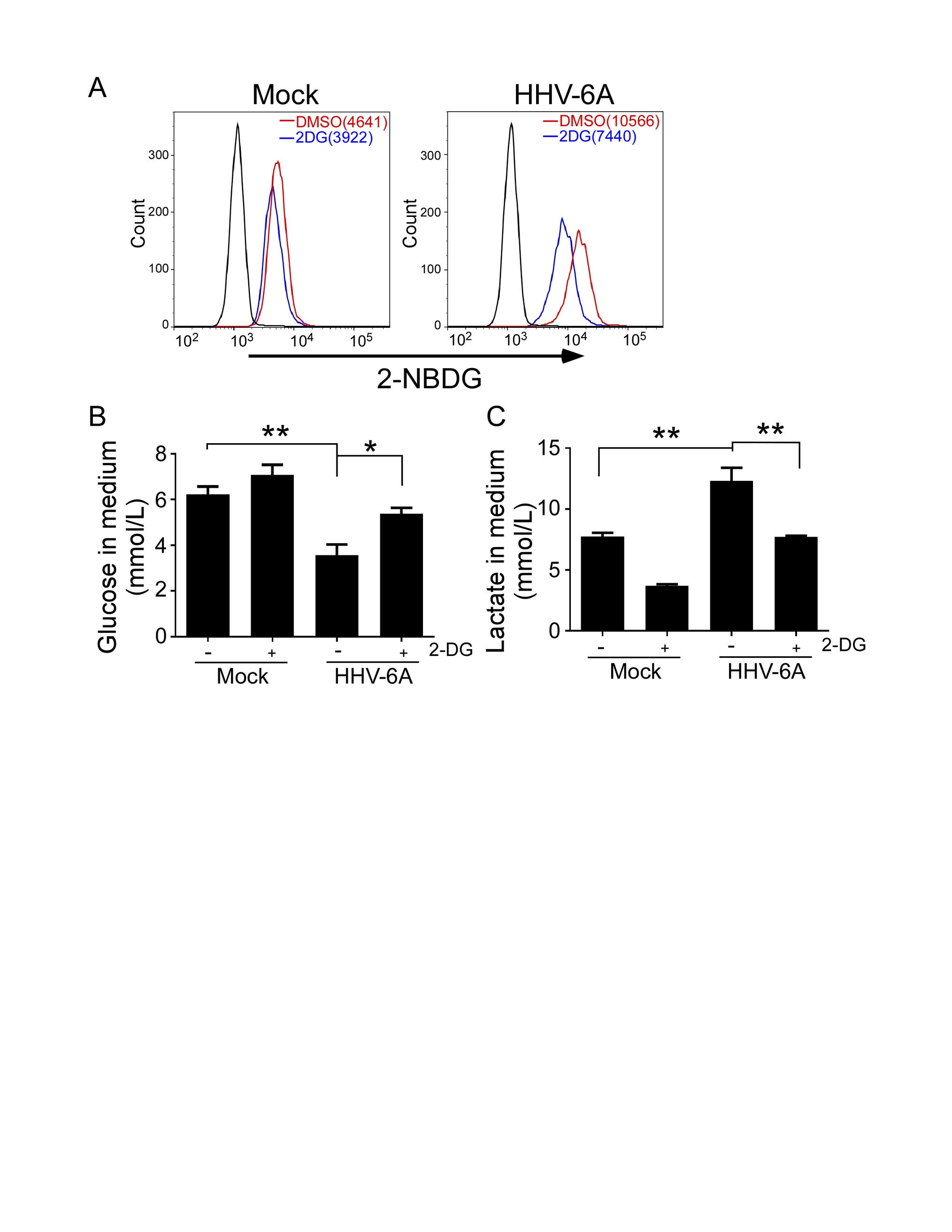

Supplement: S5 Fig — HSB-2 cells were mock infected or infected with HHV-6A. After adsorption, cells were treated with the glycolysis inhibitor 2-DG (1 mM) or DMSO. (A) 2-DG treatment significantly decreased glucose uptake in HHV-6-infected cells. Glucose uptake was determined by flow cytometry with addition of 2-NBDG for 15 min after 72 h culture. (B) 2-DG treatment increased glucose levels in the culture medium of HHV-6A infected HSB-2 cells. The glucose levels in the culture medium were determined after 72 h culture using a Glucose Oxidation Assay Kit. Results shown in histogram are mean ± SD from three independent experiments. * p<0.05, ** p<0.01, compared with the indicated control group. (C) 2-DG treatment decreased lactate secretion of HSB-2 cell. The lactate levels in culture supernatant was analyzed at 72 h post infection. Results shown in the histogram are mean ± SD from three independent experiments. ** p<0.01, compared with the indicated control group. (TIF) [file ppat.1008568.s005.tif]
