# Supplementary material for: Human herpesvirus 6A promotes glycolysis in infected T cells by activation of mTOR signaling
Source: PLoS Pathog. 2020 Jun 9;16(6):e1008568. doi: 10.1371/journal.ppat.1008568 (PMC7282626; doi:10.1371/journal.ppat.1008568)
Supplement: S1 Table — (DOCX) [file ppat.1008568.s006.docx]

**S1 Table. Primers used for real-time quantitative RT- PCR (Glycolytic enzymes)**

| Genes | Primers |
| --- | --- |
| Glut1 Forward | ATTGGCTCCGGTATCGTCAAC |
| Glut1 Reverse | GCTCAGATAGGACATCCAGGGTA |
| Glut3 Forward | GCTGGGCATCGTTGTTGGA |
| Glut3 Reverse | GCACTTTGTAGGATAGCAGGAAG |
| HK2 Forward | AACAGCCTGGACGAGAGCAT |
| HK2 Reverse | GCCAACAATGAGGCCAACTT |
| GPI Forward | GATGGTAGCTCTCTGCAGCC |
| GPI Reverse | GCCATGGCGGGACTCTTG |
| PFK1 Forward | GGCAGCCATGCATAAAGACG |
| PFK1 Reverse | AAGCTTCCCCAGCTGTTCTC |
| TPI1 Forward | CTCATCGGCACTCTGAACG |
| TPI1 Reverse | GCGAAGTCGATATAGGCAGTAGG |
| ENO1 Forward | CGCCTTAGCTAGGCAGGAAG |
| ENO1 Reverse | GGTGAACTTCTAGCCACTGGG |
| PKM2 Forward | ACGAGAACATCCTGTGGCTG |
| PKM2 Reverse | AGGAAGTCGGCACCTTTCTG |
| LDHα Forward | AGCTGTTCCACTTAAGGCCC |
| LDHα Reverse | TGGAACCAAAAGGAATCGGGA |
| β-actin Forward | TGGCACCCAGCACAATGAA |
| β-actin Reverse | CTAAGTCATAGTCCGCCTAGAAGCA |
