# Supplementary material for: Human herpesvirus 6A promotes glycolysis in infected T cells by activation of mTOR signaling
Source: PLoS Pathog. 2020 Jun 9;16(6):e1008568. doi: 10.1371/journal.ppat.1008568 (PMC7282626; doi:10.1371/journal.ppat.1008568)
Supplement: S2 Table — (DOCX) [file ppat.1008568.s007.docx]

**S2 Table. Primers used for quantitative PCR (HHV-6 U22)**

| Genes | Primers |
| --- | --- |
| β-actin Forward | TCCACCGCAAATGCTTCTAG |
| β-actin Reverse | TGCTGTCACCTTCACCGTTC |
| HHV6-U22 Forward  HHV6-U22 Reverse | CGCTCGGAAAGGAAACATTA  AAGTGGAACTGCTTGGTGGC |
